# Terminal Uridylyltransferases TUT4/7 Regulate microRNA and mRNA Homeostasis

**DOI:** 10.3390/cells11233742

**Published:** 2022-11-23

**Authors:** Pengcheng Zhang, Mallory I. Frederick, Ilka U. Heinemann

**Affiliations:** Department of Biochemistry, University of Western Ontario, 1151 Richmond Street, London, ON N6A 5C1, Canada

**Keywords:** RNA degradation, uridylation dependent decay, microRNA homeostasis, transcriptome, miRNA/mRNA network, oncogenic signaling

## Abstract

The terminal nucleotidyltransferases TUT4 and TUT7 (TUT4/7) regulate miRNA and mRNA stability by 3′ end uridylation. In humans, TUT4/7 polyuridylates both mRNA and pre-miRNA, leading to degradation by the U-specific exonuclease DIS3L2. We investigate the role of uridylation-dependent decay in maintaining the transcriptome by transcriptionally profiling TUT4/7 deleted cells. We found that while the disruption of TUT4/7 expression increases the abundance of a variety of miRNAs, the let-7 family of miRNAs is the most impacted. Eight let-7 family miRNAs were increased in abundance in TUT4/7 deleted cells, and many let-7 mRNA targets are decreased in abundance. The mRNAs with increased abundance in the deletion strain are potential direct targets of TUT4/7, with transcripts coding for proteins involved in cellular stress response, rRNA processing, ribonucleoprotein complex biogenesis, cell–cell signaling, and regulation of metabolic processes most affected in the TUT4/7 knockout cells. We found that TUT4/7 indirectly control oncogenic signaling via the miRNA let-7a, which regulates AKT phosphorylation status. Finally, we find that, similar to fission yeast, the disruption of uridylation-dependent decay leads to major rearrangements of the transcriptome and reduces cell proliferation and adhesion.

## 1. Introduction

Given the tight regulation of RNA present in the cell at any given time, it should come as no surprise that RNA degradation is comprised of numerous distinct pathways. Although the deadenylation of mRNA transcripts was once thought to be their primary degradation signal, more recent studies have pointed toward additional uridylation-dependent degradation pathways that may be key to various regulatory processes [1]. The 3′ end RNA uridylation is catalysed by terminal uridylyltransferases (TUTs), members of the terminal nucleotidyltransferase (TENT) family. Terminal uridylyltransferase activity was first discovered in *Schizosaccharomyces pombe*, where caffeine-induced death protein 1 (CID1) catalyses mRNA uridylation, marking transcripts for subsequent degradation by the U-specific DIS3-like exonuclease 2 (DIS3L2) [2]. Notably, studies in *S. pombe* identified the magnitude of mRNA uridylation present and normally occurring in cells [3], where the disruption of this pathway leads to the major rearrangement of the transcriptome and elicits a cellular stress response [4]. In human cells, one of the first noted incidences of mRNA uridylation was in histone mRNAs, where transcripts are polyuridylated for degradation during cell cycle turnover [5].

**Figure 1:**
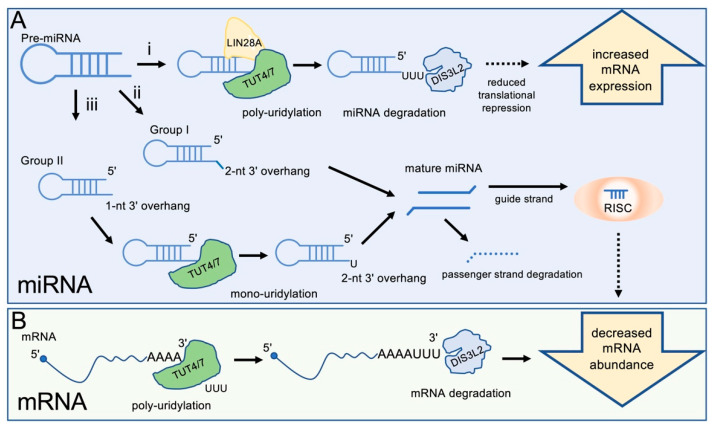
TUT4/7 regulate miRNA and gene expression levels. (**A**) miRNAs are regulated by TUT4/7 in multiple pathways: (i) Pre-miRNAs can be polyuridylated by TUT4/7 in the presence of LIN28A, a signal for degradation by DIS3L2. Decreased miRNA levels due to DIS3L2-mediated degradation increase target mRNA levels. (ii) Group I pre-miRNAs contain a 2-nucleotide 3′ overhang and can be processed by DICER to form the mature miRNA. This process occurs independently of TUT4/7 regulation of miRNAs. (iii) Group II pre-miRNAs contain a 1-nucleotide 3′ overhang and must be extended by one nucleotide at the 3′ end to form the necessary 2-nucleotide 3′ overhang for DICER processing. Mature miRNAs are processed into a guide (usually 5p) strand, which is incorporated into the RNA-induced silencing complex (RISC), and a passenger (usually 3p) strand, which is degraded. Mature miRNAs reduce expression of target mRNAs. (**B**) mRNAs can be 3′ polyuridylated by TUT4/7 and degraded by DIS3L2, decreasing gene expression.

The list of mRNA species regulated by uridylation-dependent decay pathways has grown rapidly in recent years. In humans, two largely redundant enzymes, TUT4 (ZCCHC11/TENT3A) and TUT7 (ZCCHC6/TENT3B), target a diverse group of RNAs, including ncRNAs- and mRNA-derived fragments [6], influenza A virus mRNAs [7], and LINE-1 retrotransposons [8], among many others (Figure 1). RNA specificity of TUT4/7 is thought to be largely determined by RNA-binding proteins, such as the Lin-28 Homolog A or B (LIN28A/B), which facilitate TUT4/7 binding to RNA [9,10]. In breast cancer models, TUT4 inhibition has preventative effects on tumour formation [11], making it a potential chemotherapy target. The knockdown of TUT4 leads to decreased tumour volume in cells expressing high levels of the RNA-binding protein LIN28A, a common feature of HER2+ cancers [11]. Interestingly, the same study showed that TUT4 knockdown had little effect on triple negative breast cancers, where LIN28B is highly expressed [11]. This difference may point toward the distinct roles of TUT4/7 uridylation in target-specific contexts or may be reflective of cell type-specific differences. Regardless, studies, such as this one, are of importance as TUT4/7 inhibitors are increasingly available, making the enzymes promising chemotherapeutic targets.

TUT4/7 have been implicated in global mRNA turnover events during apoptosis [12] and hundreds of genes have been identified with decreased uridylation upon their knockdown (Figure 1) [13]. TUT4/7 are associated with global transcriptome turnover of maternal mRNA, a conserved role found in *Xenopus*, zebrafish, and mammals [14,15]. During the early stages of the maternal-to-zygotic transition, TUT4/7 proteins are translated from maternal mRNA; their increased expression drives uridylation and subsequent degradation of maternal transcripts by DIS3L2, paving the way for the increased expression of proteins translated from zygotic mRNAs [14]. In mice, the conditional knockout of these two proteins revealed a role in fertility, where TUT4/7 catalytically deficient cells cannot properly complete meiosis [15]. These data revealed an underlying link between TUT4/7 and infertility, in addition to the cells’ inability to overhaul their transcriptome during early development.

Although TUT4/7 and DIS3L2 are primarily recognized for their respective roles in mRNA uridylation and degradation, these enzymes are further associated with microRNA (miRNA) maturation, degradation, and stability. In miRNA metabolism, the role of uridylation by TUT4/7 in the cell is multifaceted, and several studies show that TUT4/7 can alter between processive oligouridylation and distributive monouridylation [16,17]. As with mRNA degradation, oligouridylation of pre-miRNAs by TUT4/7, in collaboration with the RNA-binding protein LIN28A, marks the miRNAs for degradation by the U-specific exonuclease DIS3L2 [16]. Whereas DIS3L2 degrades polyuridylated miRNAs [18,19,20], TUT4/7 are also capable of monouridylating miRNA substrates, which is generally associated with the stabilization of group II pre-miRNAs and increased mature miRNA levels [21]. During the maturation of group II miRNAs, TUT4/7 add a single uridine residue to the pre-miRNA substrate, a prerequisite to further processing by DICER [1,21]. These monouridylated miRNAs include the precursors of various let-7 family miRNAs [10,21,22], pre-miR-26a and mature miR-191 [16], and pre-miRNAs associated with AGO2 (argonaute RISC catalytic component (2)) during miRNA biogenesis [20,23]. Despite the contrasting outcomes of mono- and polyuridylation by TUT4/7, the knockdown of TUT4/7 leads to increased levels of mature group II miRNAs, along with decreased levels of their mRNA targets (Figure 1) [10,23].

It is thought that the switch between mono- and polyuridylation is dependent on RNA-binding proteins. As in the case of mRNAs, the RNA-binding protein LIN28A recruits TUT4 to miRNA substrates and triggers polyuridylation activity, reducing mature miRNA levels via uridylation-dependent degradation (Figure 1) [11,24]. This mechanism is similarly reflected in other TENT family members, where the RNA-binding protein Quaking-7 (QKI-7) directs TENT2 (Gld2, PAPD4) RNA specificity and adenylyltransferase activity [25]. More recently, monouridylation by TUT4/7 was shown to alter target specificity of miRNAs by adding a single uridine to the mature miRNA 3′end. This monouridylation leads to tail-U-mediated repression (TUMR) by modifying the pool of potential target mRNAs [26].

Our group previously used *S. pombe* deletion models to investigate the roles of CID1, a TUT4/7 homolog, and DIS3L2 in vivo [4]. We identified a global role for these proteins in mRNA turnover, where ΔCID1 cells were deficient in uridylated transcripts and had increased sensitivity to cell stress, including oxidative stress and protein misfolding. Genes differentially regulated in ΔCID1 cells overlapped significantly with those differentially regulated in ΔDIS3L2 cells, with shared transcripts representing a third of those differentially expressed in the ΔCID1 strain alone [4]. This global analysis highlights the major roles of these uridylation-dependent pathways in cellular homeostasis. Here, we use a HEK 293T ΔTUT4/7 cell line to characterize the extent of TUT4/7 uridylation in cells. Using a combination of mRNA-seq, miRNA-seq, and standard biochemical procedures, we show that HEK 293T ΔTUT4/7 cells have upregulated the abundance of genes involved in stress response pathways and increased levels of mature miRNAs, with many of these changes reflected at the protein level. Additionally, HEK 293T ΔTUT4/7 cells exhibit significant growth defects and reduced ability to adhere to tissue culture plastics relative to wild-type cells. Finally, TUT4/7 alter oncogenic signalling via the miRNA let-7a, which regulates phosphorylation and the activity of the oncogenic kinase AKT. Together, these results indicate substantial roles for TUT4/7 in cell signalling, cell adhesion, and cancer signalling.

## 2. Materials and Methods

### 2.1. Cell Culture

HEK 293T cells were obtained from the American Type Culture Collection (ATCC CRL-3216, American Type Culture Collection, Manassas, VA, USA). TUT4/7 double knockout (ΔTUT4/7) cells were generated and published previously and were provided as a kind gift from Dr. Gu [26]. Wild-type (WT) or TUT4/7 double knockout (ΔTUT4/7) HEK 293T cells were cultured at 37 °C with 5% CO_2_, according to ATCC Culture Guides. Dulbecco’s Modified Eagle Medium (DMEM) supplemented with 10% fetal bovine serum (FBS), 100 units/mL penicillin, and 100 µg/mL streptomycin was used as culture medium. For RNA extraction and Western blotting, cells were grown to between 70–80% confluence and harvested with phosphate-buffered saline (PBS, 137 mM NaCl, 2.7 mM KCl, 10 mM Na_2_HPO_4_, and 1.8 mM KH_2_PO_4_, pH 7.4). Cells were stored at −80 °C until further use.

### 2.2. RNA Extraction

Prior to RNA isolation, cells were cultured at 37 °C with 5% CO_2_ for 48–72 h, at 70% to 80% confluence and harvested with PBS. In brief, culture media were aspirated and cells suspended in PBS. Cells were then collected by centrifuging the PBS cell suspension at 4 °C, at 4,000× *g* for 5 min. For RNA extraction, the cells were further processed using the miRNeasy Mini Kit (Qiagen, Hilden, Germany), according to manufacturer’s instructions. Sample quality for sequencing was confirmed by bioanalyzer (Agilent Technologies, Santa Clara, CA, USA) and RNA concentration was measured on a NanoDrop 2000 spectrophotometer (ThermoFisher, Waltham, MA, USA).

### 2.3. RT-qPCR

RT-qPCR reactions were performed as described [27]. Briefly, for each reaction, 2 μg of total RNA was reverse transcribed using High-Capacity cDNA Reverse Transcription Kit (Applied Biosystems, Waltham, MA, USA). The cDNA was amplified with PowerUp or PowerTrack SYBR Green (Applied Biosystems) on QuantStudio 3 Real-Time PCR System. At least 3 biological replicates, independent of RNA-seq samples, were analyzed with 2 technical replicates. For each target, the control without reverse transcriptase (no RT control) was used to exclude false-positive amplification. Quantification was performed by ΔΔC_T_ method, using SNORD47 as reference for miRNAs and RPS18 for mRNAs, and *p*-values were calculated by Student’s *t*-test or ANOVA in GraphPad Prism. Primers are listed in supplementary Table S1.

### 2.4. Cell Proliferation Assay

Cell viability was determined by Cell Counting Kit-8 (CCK-8, Sigma-Aldrich, St. Louis, MO, USA), according to manufacturer’s instructions. Briefly, HEK 293T WT and ΔTUT4/7 cells were trypan blue stained, counted by hemocytometer, and plated at equal cell densities of 5×10^4^ cells per mL. 10 µL of CCK-8 substrate was added to three separate wells from each cell type immediately after seeding or after cells were grown for 24, 48, and 72 h. OD at 450 nm was read 4 h after the addition of CCK-8 on Synergy H1 plate reader (BioTek, Winooski, VT, USA), and values were normalized to media containing no cells. Cell images were taken at the 72 h timepoint using an EVOS AutoFL microscope (Thermofisher).

### 2.5. Cell Adhesion Assay

Cell adhesion assays were performed as described previously [28]. Briefly, WT and ΔTUT4/7 HEK293T cells were stained with trypan blue, diluted to 4 × 10^5^ cells per mL, and 100 µL of cells plated on standard 96-well tissue culture-treated plates. Cells were incubated at 37 °C with 5% CO_2_ for 30 min to allow reattachment, then washed with ice-cold DMEM. Cells were fixed with cold methanol (−20 °C) for 10 min, washed with ice-cold PBS, and stained with 0.1% crystal violet in 25% methanol for 30 min at room temperature. Stained cells were washed with deionized, distilled water, then the plate was inverted and dried. Finally, cells were dissolved in 100 μL of 1% SDS, and absorbance at 570 nm was measured on a Synergy H1 plate reader (BioTek, Winooski, VT, USA).

### 2.6. Western Blotting

Western blotting was performed as described previously [4,27]. Briefly, total protein was extracted from WT or ΔTUT4/7 HEK 293T cells at approximately 80% confluence. Cells were harvested and suspended in 200 µL RIPA lysis buffer (50 mM Tris-HCL, pH 8.0, 150 mM NaCl, 1% v/v Nonidet p-40, 0.5% Sodium Deoxycholate, 0,1% SDS) supplemented with 2 µL 30 mM phenylmethyl sulfonyl fluoride (PMSF) and protease inhibitor cocktail (Roche, Basel, Switzerland). Lysates were centrifuged at maximum speed for 10 min at 4 °C. Cell-free extracts were transferred to new microcentrifuge tubes, and the pellet was discarded. Protein concentrations were measured by BCA assay (ThermoFisher).

Amounts of 10–60 μg of protein were loaded on 8%, 10%, or 12% SDS-PAGE gel, depending on target protein molecular weight. Transfer was performed with the Trans-Blot Turbo Transfer System (Bio-Rad, Hercules, CA, USA) at 25 V, 1.3 A for 15 min. Membranes were blocked with 5% BSA in TBST for 1 h at room temperature, then incubated with primary antibody (anti-Vinculin, Millipore-Sigma (Burlington, MA, USA) #V9131; anti-HSP90, Cell Signaling Technology (Danvers, MA, USA) #4874; anti-HSP70, Invitrogen (Waltham, MA, USA) #MA3-006; anti-pan-AKT, Cell Signaling Technology #2920; anti-AKT1, Cell Signaling Technology #2938; anti-AKT2, Cell Signaling Technology #3063; anti-AKT3, Cell Signaling Technology #4059; anti-pAKT^T308^, Cell Signaling Technology #9275; anti-pAKT^S473^, Cell Signaling Technology #9271; anti-GAPDH, Millipore #MAB374) overnight at 4 °C. Membranes were washed 3 times with 1% BSA in TBST for 10 min, then incubated with secondary antibody (IRDye^®^ 800CW Donkey anti-Rabbit IgG Secondary Antibody, LI-COR #926-32213; IRDye^®^ 800CW Donkey anti-Mouse IgG Secondary Antibody, LI-COR #926-32212) in 1% BSA in TBST for 1 h at room temperature. After 3 washes in TBST for 5 min, blots were stored in TBS and imaged using LI-COR Odyssey classic (LI-COR Biosciences. Lincoln, NE, USA). Blots were quantified on ImageLab software (Bio-Rad). *p*-values were calculated by Student’s *t*-test or ANOVA in GraphPad Prism.

### 2.7. RNA Sequencing and Data Analysis

Three biological replicates of RNA samples from WT and ΔTUT4/7 HEK 293T cells were prepared. Total RNA samples were processed using VAHTS Total RNA-seq (H/M/R) Library Prep Kit for Illumina (Vazyme), including rRNA reduction or QIAseq miRNA Library Kit (Qiagen). Samples were fragmented, cDNA was synthesized, tagged, cleaned-up, and subjected to PCR with barcoded reverse primers (ScriptSeq Index PCR Primers, Qiagen) to permit equimolar pooling of samples into one library. All samples were sequenced at the London Regional Genomics Centre (Robarts Research Institute, London, Ontario, Canada) using the Illumina NextSeq 500 (Illumina Inc., San Diego, CA, USA). The libraries were sequenced as a paired-end run, 2 × 76 bp, using a High Output v2 kit (150 cycles). RNA sequencing data were processed using Partek^®^ Flow^®^ software v10.0.22 (Partek, Chesterfield, MO, USA). The mRNA sequencing reads were aligned to the human reference genome hg38 using STAR 2.7.3a and annotated with Ensembl Transcripts release 102. The miRNA sequencing reads were trimmed to 18 bp in length, aligned to hg38 using Bowtie 1.0.0, and annotated with hg38-miRbase mature microRNAs version 22 deduplicated. Features were excluded if maximum reads were ≤ 12.0 or 7.0 for mRNA or miRNA, respectively. Reads of both mRNA and miRNA were normalized using counts per million and adding 1.0. Fold changes (FC) and false discovery rates were calculated using Partek’s gene-specific analysis (GSA). FC values are graphed as (log2 FC), and −1/+1 cut-off was used for significance.

The Search Tool for the Retrieval of Interacting Genes/Proteins (STRING) database [29] was used to analyse protein–protein interaction (PPI) networks based on overabundant mRNAs. For downregulated mRNAs, more stringent parameters were applied to reduce the number of nodes, and an FDR < 1^−10^; FC ≥ 2, yielding 407 genes; and minimum required interaction score of 0.5 were applied; unconnected nodes were hidden. Strength values in STRING analysis were calculated by log10 (observed/expected), describing how large the enrichment effects are. The colouring of displayed STRING networks was carried out by selecting the biological processes with the GO terms with the lowest FDR values. For GO analysis, significantly regulated gene sets (FDR < 0.05, FC ≥ 2) were tested by GO PANTHER, and only GO terms with FDR < 0.05 were selected. The fold enrichment value of a GO term was obtained through comparing the background frequency of total genes annotated to the GO term to the sample frequency representing the number of probed genes that fall under the same term [30].

The miRNA–mRNA network was constructed using the miRNet database [30]. The mRNA targets of experimentally validated miRNA-target interactions were imported from miTRarBase 8.0 [31]. Upregulated let-7 miRNAs were entered as queries and manual-batch selection was used to display only significantly downregulated gene targets (FDR < 0.05, FC ≤ 2) on the network. The group II miRNA list was built using the MirGeneDB 2.1, where they were denoted as “yes” in the attribute of 3′ NTU of the human miRNA gene database [32]. The miRNA family analysis was performed using TAM 2.0 [33].

## 3. Results

Since the discovery of uridylation-dependent RNA decay, TUT4/7 have been associated with diverse roles in mRNA and miRNA turnover [1]. However, the global regulatory network is yet to be elucidated. We, therefore, investigated the impact of a deletion of both enzymes on the mRNA transcriptome and miRNome.

### 3.1. TUT4/7 Regulate miRNA Abundance

**Figure 2:**
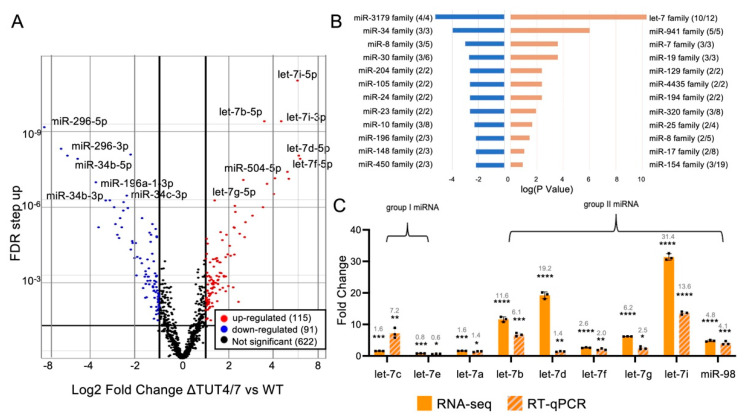
MicroRNAs are differentially expressed in HEK 293T cells lacking TUT4/7. (**A**) Volcano plot of miRNA expression plotting log2 fold change, in ΔTUT4/7 relative to wild-type HEK 293T cells, versus false discovery rate (FDR). Significantly (FDR ≥ 1, log2FC ≥ 1 or ≤ −1) changed miRNAs are indicated in red (upregulated, 115 miRNAs),blue (downregulated, 91 miRNAs) or not significantly changed miRNAs in black (622 miRNAs). (**B**) Bar graph representing the results of a microRNA family set analysis using TAM2.0, showing numerous miRNA families are enriched in significantly up- (orange) and downregulated (blue) miRNAs. Enrichment is expressed as log (*p* value) of the significance for miRNA enrichment. (**C**) RNA sequencing (orange) and RT-qPCR (orange and grey hashmarks) analysis reveal the different regulatory patterns between group I and group II let-7 miRNAs in ΔTUT4/7 cells: group I let-7 miRNAs have relatively small changes in expression in ΔTUT4/7 cells, while most group II let-7 miRNAs are drastically upregulated. Let-7a is encoded at multiple genomic locations as both group I and group II pre-miRNAs. All fold change values are shown relative to wild-type miRNA expression levels. Significance was calculated by Partek gene-specific analysis (GSA) (RNA-seq) or Student’s *t*-test (RT-qPCR) and is indicated by asterisks, where * *p* < 0.05, ** *p* < 0.01, *** *p* < 0.001, **** *p* < 0.0001.

TUT4/7 regulate miRNAs by adding untemplated uridines to the 3′ end. Although the distinct roles of TUT4/7 have been well defined, it is unclear to what extent TUT4/7 regulate overall miRNA abundance. To investigate the regulation of miRNAs by these two enzymes, we performed miRNA sequencing of HEK 293T cells, comparing a TUT4/7 knockout strain [26] (ΔTUT4/7) to wild type cells (WT). We analysed miRNA expression using Partek gene-specific analysis (GSA). In total, we identified 115 miRNAs that increased in abundance by more than 2-fold in ΔTUT4/7 cells and 91 miRNAs that decreased by at least 2-fold (Figure 2A, Tables S2 and S3, Supplementary Files S1–S3). For 27 miRNA pairs, both the 5′ strand and the 3′ strand were detected. In general, both the 5p and 3p miRNAs from the same pre-miRNA increased or decreased similarly, for example, let-7i-5p levels increased by 31-fold in ΔTUT4/7 cells, while let-7i-3p increased by 33-fold (Figure 2A, Table S4). However, for the let-7 family miRNA, in particular, the 5p miRNAs are thought to act as the guide strand, while the 3p miRNA is degraded [34]. We, therefore, focused our further analysis on 5p miRNAs when both miRNA strands were detected, unless otherwise specified.

#### 3.1.1. Several miRNA Families Are Increased in Abundance in ΔTUT4/7 Cells

Our sequencing data show that 115 miRNA species increased in abundance upon TUT4/7 deletion (Table S2, Supplementary Files S2 and S3). Interestingly, these miRNAs are found in specific miRNA families rather than random enrichment. Members of the let-7 miRNA family are among the miRNAs most increased in abundance, with 10 of 12 let-7 family miRNAs increased by at least 2-fold (Figure 2B, Table S5, Supplementary Files S5 and S6). The tumour suppressor let-7 miRNAs have previously been identified as TUT4/7 substrates [9,18,21], and a recent study similarly identified increased abundance of these miRNAs upon TUT4/7 deletion [35]. In addition, all members of the following miRNA families significantly increased in abundance by at least 2-fold: miR-941, miR-7, miR-19, miR-129, miR-4435, and miR-194 (Figure 2B). These miRNA families are generally involved in cell proliferation and differentiation and can play either tumour suppressor or oncogenic roles: the oncogenic human-specific miR-941 is suggested to play a role in cell differentiation, with high expression in pluripotent cells and decreased expression during cell differentiation [36], while the tumour suppressors miR-7 and miR-194 are thought to be involved in the regulation of metastasis and cell proliferation, where high miRNA levels reduce cancer metastasis [37,38]. Some upregulated miRNA families have additional roles: miR-19 family miRNAs drive cell differentiation in neuronal cells [39], and miR-129 family members can function as both tumour suppressors and oncogenes [40]. Finally, miR-4435 miRNAs are thought to be excreted and function as circulating oncogenic miRNAs [41]. These data demonstrate the diversity and multitude of TUT4/7-regulated miRNAs and their functions.

#### 3.1.2. The miRNA Families Decreased in Abundance in ΔTUT4/7 Cells

Our sequencing data show 91 miRNA species with abundance significantly decreased by at least 2-fold in ΔTUT4/7 cells (Figure 2A, Table S3, Supplementary Files S1, S3 and S7). As in the overrepresented miRNAs, the underrepresented miRNAs cluster into several miRNA families rather than a random distribution, indicating family-specific miRNA regulation by TUT4/7 (Figure 2B, Table S6). For example, the miR-34 family of tumour suppressor miRNAs [42] were significantly underrepresented: miR-34a, miR-34b, and miR-34c were 4.8-, 31.6-, and 63.2-fold decreased in abundance, respectively, in ΔTUT4/7 cells. We also found significant downregulation of miR-125b-1, miR-125b-2, and miR-99a from the miR-10 family (three out of eight), along with miR-30a, miR-30c-1, and miR30c-2 from the miR-30 family (three out of six) (Figure 2B, Table S3). Both the miR-10 and miR-30 families are established tumour suppressor miRNAs, with roles in the regulation of cell proliferation, apoptosis, cell cycle, migration, invasion, and metastasis [43,44].

#### 3.1.3. TUT4/7 Deletion Increases Expression of Group II miRNAs

As outlined above, group II miRNAs undergo a maturation step wherein TUT4/7 add a single nucleotide to the 3′ end of the pre-miRNA as a prerequisite to DICER processing (Figure 1). In addition, TUT4/7 polyuridylate miRNAs, leading to miRNA degradation. Since group II miRNAs require nucleotide addition for maturation, it is possible that TUT4/7 deletion affects group II miRNAs differently than group I miRNAs. Here, if TUT4/7 were essential for group II miRNAs, the deletion should cause a significant reduction in mature group II miRNAs. A total of 45 miRNAs are defined as group II miRNAs [16,21,32], of which we found 20 up- and 7 downregulated miRNAs (at least 2-fold, FDR < 0.05, Table S7, Supplementary File S8) in ΔTUT4/7 cells, indicating that TUT4/7 are not essential for group II miRNA maturation and not all group II miRNAs are affected in the same way.

We decided to investigate the let-7 miRNA family more closely, which is comprised of both group II and group I miRNAs. Most let-7 family miRNAs have similar functions as tumour suppressor miRNAs. To verify our sequencing data, we quantified the change in let-7 family miRNA abundance using RT-qPCR (Figure 2C). We found that RT-qPCR data generally agreed with the fold change in the sequencing data, even if the absolute value of the magnitude of change varied (Figure 2C). Among the let-7 family, we found the group II miRNAs let-7b, let-7d, let-7f, let7-g, let-7i, and miR-98 had increased abundance in ΔTUT4/7 cells, with the highest increase being over 30-fold for let-7i (Figure 2C). If group II miRNAs relied solely on TUT4/7 for maturation, a decrease in mature miRNAs would be expected in knockdown cells. Our data indicate that although TUT4/7 were previously shown to play a role in group II miRNA processing, this role can be fulfilled by other TENT enzymes. For example, TENT2 is thought to monoadenylate group II miRNAs in lieu of TUT4/7 [45,46], and that role was recently investigated [35]. In the case of group I miRNAs (let-7c and let-7e), expression levels in ΔTUT4/7 cells remained stable, with less than 2-fold changes in abundance (Figure 2C). Finally, let-7a, which is present in three copies in the human genome (let-7a-1, let-7a-2, let-7a-3), is processed from both group I and group II pre-miRNAs and remained mostly stable in abundance, with a slight 1.6-fold increase in abundance in ΔTUT4/7 cells (Figure 2C). Although monouridylation was previously shown to play a major role in determining non-canonical miRNA target recognition [26] and the generation of 3′ isomiRs [35], monouridylation of group II precursor miRNAs may be considered a minor, non-essential role of TUT4/7, as group II miRNA family members are still processed to maturity in the absence of TUT4/7.

### 3.2. TUT4/7 Are Global Regulators of Gene Expression Levels

**Figure 3:**
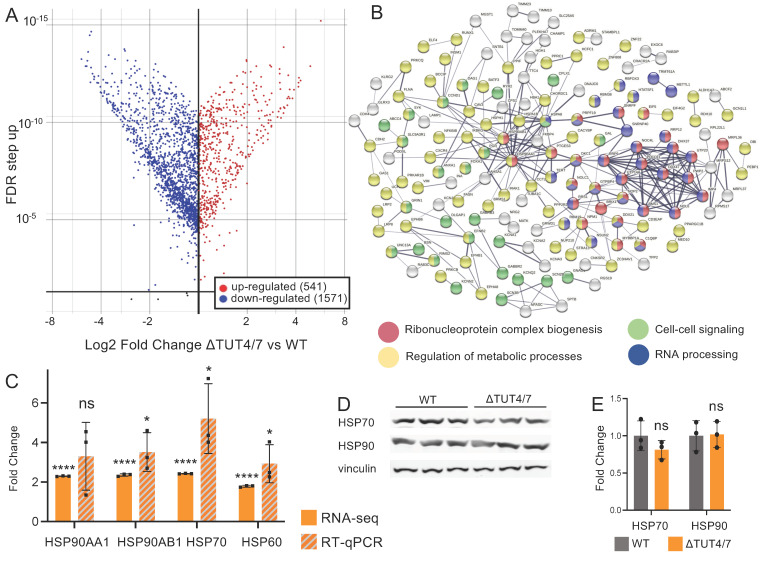
Messenger RNAs are differentially regulated in HEK 293T cells lacking TUT4/7. (**A**) Volcano plot of mRNA log2 expression fold change (FC) in ΔTUT4/7, relative to wild-type HEK 293T cells, versus false discovery rate (FDR). Significantly (FDR ≥ 1, log2FC ≥ 1 or ≤ −1) changed mRNAs are indicated in red (upregulated, 541 mRNAs) or blue (downregulated, 1573 miRNAs). The mRNAs not significantly changed in expression are depicted in black. (**B**) Search Tool for the Retrieval of Interacting Genes/Proteins (STRING) analysis reveals a physical subnetwork of mRNAs that are at least 2-fold upregulated in ΔTUT4/7 cells. GO analysis of the STRING network shows enrichment in the following: ribonucleoprotein complex biogenesis (red, strength 0.53), regulation of metabolic process (yellow, strength 0.12), cell–cell signaling (green, strength 0.27), and RNA processing (blue, strength 0.29). Differentially expressed mRNAs not included in these GO terms are indicated in white; those with no interactions to others are not included in the network. Confidence strength of data support was calculated by log10 (observed/expected) and is indicated by line thickness, with thicker lines indicating stronger supporting data. (**C**) RNA sequencing (orange) and RT-qPCR (orange and grey hashmarks) of mRNAs involved in heat shock response in ΔTUT4/7 cells. Fold change is calculated relative to wild-type HEK 293T cells. (**D**) Western blotting and (**E**) quantification of heat shock response protein levels in WT (grey) and ΔTUT4/7 (orange) HEK 293T cells. Significance was calculated by Partek gene specific analysis (GSA) (RNA-seq) or Student’s *t*-test (RT-qPCR, Western blot) where ns = not significant; * *p* < 0.05,**** *p* < 0.0001.

In addition to miRNAs, many mRNAs are well-recognized TUT4/7 substrates and can be degraded by uridylation-dependent decay [1]. To further assess the impact of a TUT4/7 knockout on gene expression levels, we carried out a transcriptomic analysis using Illumina sequencing. We found 2132 differentially expressed genes (DE-mRNAs) that are significantly changed by at least 2-fold in abundance, among which 541 are upregulated and 1571 are downregulated (Figure 3A, Supplementary Files S9–S11). If the changes in gene expression levels are TUT4/7-dependent, direct mRNA substrates of TUT4/7 would be expected to increase in abundance in the knockout cell line, where uridylation-dependent RNA decay is perturbed [4]. In contrast, the abundance of mRNAs impacted by the change in miRNA abundance are predicted to decrease due to increased miRNA abundance. Although we expect that some changes in gene expression levels may be due to secondary effects, such as changes in expression to compensate for the loss of uridylation-dependent decay, or the activation of the cellular stress response as seen in yeast [4], we proceeded to analyse our data by categorizing the differentially abundant mRNAs into two groups for further analysis: (1) mRNAs upregulated in the knockout strain and (2) mRNAs downregulated in the TUT4/7 knockout.

#### 3.2.1. Messenger RNAs Can Be Upregulated in ΔTUT4/7 Cells

Given that direct targets of TUT4/7 should not be subjected to uridylation-dependent decay in ΔTUT4/7 cells, we deduce that mRNAs with increased abundance in the knockout strain are likely direct targets of TUT4/7. We, therefore, analyzed whether mRNAs with increased abundance are clustered into specific pathways. Proteins corresponding to mRNAs with increased abundance were analysed using the Search Tool for the Retrieval of Interacting Genes/Proteins (STRING) database to probe for physical protein interaction (Figure 3B) [29]. We also analysed the mRNA datasets using gene ontology (GO) analysis to identify biological processes and molecular functions impacted by the TUT4/7 deletion (Table S8) [47,48]. We found most of these overabundant transcripts are involved in regulatory pathways, with specific clusters belonging to ribonucleoprotein complex biogenesis (Figure 3B, red, strength 0.53), regulation of metabolic process (Figure 3B, yellow, strength 0.12), cell–cell signaling (Figure 3B, green, strength 0.27), and RNA processing (Figure 3B, blue, strength 0.29) (detailed in Table S8). These data show that TUT4/7 control gene abundance in many cellular processes and are global regulators of mRNA homeostasis in mammalian cells.

Since the heat stress response was one of the most significantly upregulated pathways in the GO analysis (Table S7) and was also significantly increased in our previous studies in the *S. pombe* ΔCID1 strain [4], we decided to further investigate the heat stress response in ΔTUT4/7. We probed four transcripts that translate to three HSP proteins. HSPA70 (heat shock protein family A member 80), HSP60 (gene: heat shock protein family D member 1), and HSP90, which is encoded by two genes: HSP90AA1 (heat shock protein 90 alpha family A class member 1) and HSP90AB1 (heat shock protein 90 alpha family class B member 1). First, we validated selected sequencing data using RT-qPCR for transcripts (Figure 3C). Similar to the disruption of uridylation-dependent decay in *S. pombe*, chaperones, including heat shock proteins HSP90, HSP70, and HSP60, are significantly upregulated at the mRNA level (Figure 3C) in our sequencing analysis. The significant upregulation was confirmed by RT-qPCR, except for one of the HSP90 homologs, HSP90AA1, where no significant upregulation was observed. This is a surprising contrast to previous studies, which showed HSP90AA1 to be the inducible HSP90 homolog, while HSP90AB1 is thought to be constitutively expressed [49]. Our sequencing data indicated increases of 2.3-fold for HSP90AA1 and 2.4-fold for HSP90AB1, yet Western blotting showed no change in HSP90 protein levels (Figure 3D,E). The mRNAs encoding HSP70 and the chaperoning HSP60 are similarly increased by 2.4- and 2-fold, respectively, in ΔTUT4/7 cells (Figure 3C), yet protein levels of HSP70 did not change (Figure 3D,E). These data indicate that although gene expression levels for chaperones are increased, their expression is regulated at a posttranscriptional level to keep protein abundance unchanged.

#### 3.2.2. Messenger RNAs Can Be Downregulated in ΔTUT4/7 Cells

**Figure 4:**
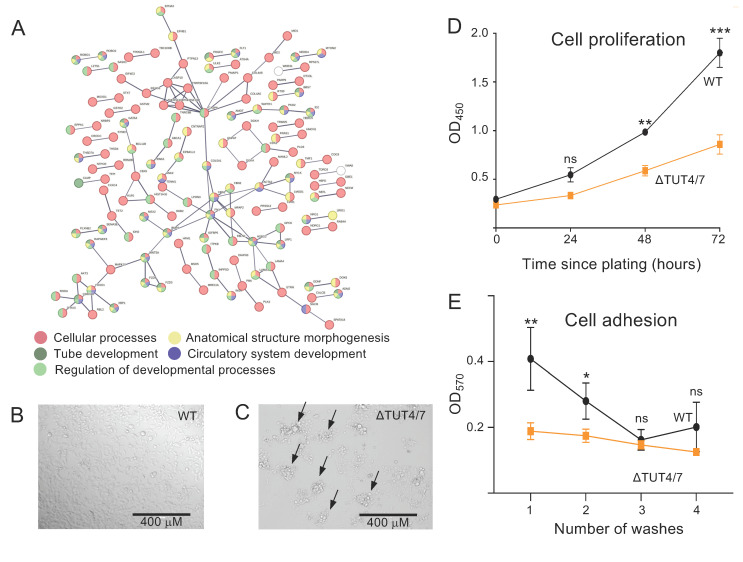
Messenger RNAs downregulated in ΔTUT4/7 cells are implicated in changes to cellular phenotype. (**A**) Search Tool for the Retrieval of Interacting Genes/Proteins (STRING) analysis reveals a physical subnetwork of mRNAs that are at least 2-fold downregulated in ΔTUT4/7 cells with a stringent false discovery rate to reduce nodes (FDR < 1^−10^). Gene Ontology (GO) analysis of the STRING network shows enrichment of cellular processes (red, strength 0.06), circulatory system development (blue, strength 0.42), regulation of developmental processes (light green, strength 0.26), anatomical structure morphogenesis (yellow, strength 0.28), and tube development (dark green, strength 0.41). Differentially expressed mRNAs not included in these GO terms are indicated in white; those with no interactions to others are not included in the network. Confidence strength of data support is calculated by log10 (observed/expected) and indicated by line thickness, with thicker lines indicating stronger supporting data. (**B**,**C**) Representative images of (**B**) wild-type and (**C**) ΔTUT4/7 HEK 293T cells 72 h after plating at 5 × 10^4^ cells per mL show multicellular aggregates in ΔTUT4/7 cells (black arrows). (**D**) CCK-8 cell proliferation assay of wild-type (black circles) versus ΔTUT4/7 (orange squares) HEK 293T cells. (**E**) Quantification of a crystal violet cell adhesion assay of wild-type (black circles) versus ΔTUT4/7 (orange squares) HEK 293T cells. Significance was calculated by 2-way ANOVA in GraphPad Prism and is indicated by asterisks, where ns = not significant; * *p* < 0.05; ** *p* < 0.01; *** *p* < 0.001.

Although the deletion of TUT4/7 in many cases increases gene expression levels, as outlined above, we found that 1571 mRNAs decreased by at least 2-fold in abundance (Figure 3A). The fact that almost 3-fold more mRNAs are decreased in abundance than increased indicates an extensive indirect regulation of mRNA levels. Interestingly, STRING analysis reveals a physical subnetwork of mRNAs that are at least 2-fold downregulated in ΔTUT4/7 cells, with functions in cell morphogenesis and cell adhesion and developmental processes (Figure 4A, Table S9). Similarly, GO terms related to growth factor signalling pathways are enriched in the decreased-abundance mRNAs (GO:0040036, 4.6-fold enrichment, Table S9), as well as cell migration pathways (GO:0001755, 3.6-fold enrichment; GO:0090497, 3.5-fold enrichment; GO 2000177, 3.3-fold enrichment, Table S9). Finally, pathways related to cell adhesion were decreased in ΔTUT4/7 cells (GO:0007160, 2.5-fold enrichment; GO:0001763, 2.5-fold enrichment, Table S9). For example, HAND1, PCDH8, and 77 other downregulated mRNAs are related to the GO term “tissue morphogenesis” (77/560); DDR1, COL13A1, and 23 other downregulated mRNAs are related to the GO term “cell-matrix adhesion” (23/133).

#### 3.2.3. TUT4/7 Deletion Alters Cellular Phenotypes

Guided by our STRING and GO analyses, we decided to carry out a phenotypic characterization of ΔTUT4/7 cells. Indeed, cell imaging revealed an altered adhesion of cells to tissue culture plates (Figure 4B,C), where ΔTUT4/7 formed multicellular aggregates after 72 h rather than a monolayer, indicating a disruption in cell adhesion. ΔTUT4/7 cells also showed reduced cell proliferation rates relative to WT HEK 293T (Figure 4D). As cellular aggregates in cell culture are often associated with reduced adhesion to the tissue culture plates [50,51], we conducted a crystal violet-based cell adhesion assay [28]. Here, ΔTUT4/7 cells showed significantly reduced adhesion to tissue culture plates, with most cells dislodged from the tissue culture plates in the first wash cycle (Figure 4E). These phenotypic data confirm that the changes on the mRNA level in cell cycle progression and cell adhesion manifest in reduced cell adhesion and proliferation.

### 3.3. The miRNA–mRNA Network in ΔTUT4/7 Cells

**Figure 5:**
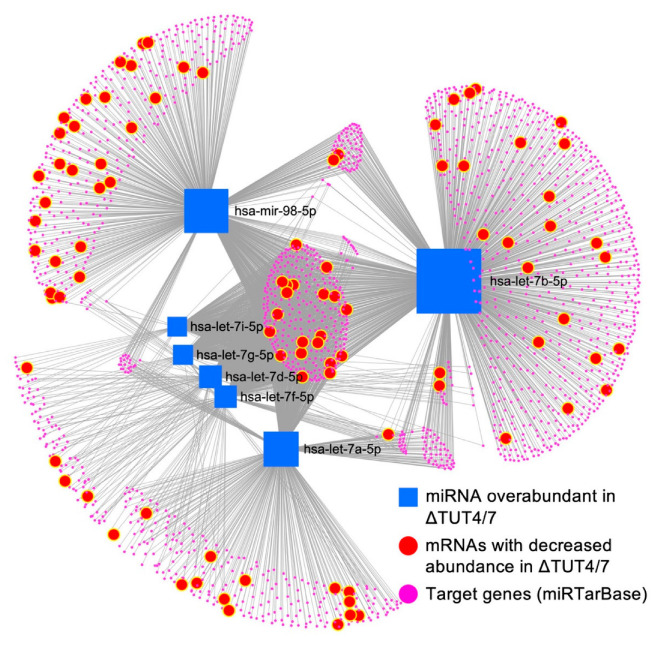
The miRNA–mRNA interaction network reveals target distribution among 1933 target mRNAs (pink circles) of the upregulated let-7 miRNAs (blue squares), 94 are downregulated at least two-fold (highlighted in bigger red circles) in ΔTUT4/7 HEK 293T cells. The miRNA–target interaction is based on experimentally validated information obtained from miRTarbase v8.0. The diagram was generated using miRNet 2.0.

TUT4/7 deleted cells are revealed to have a 3-fold larger amount of downregulated genes, compared to upregulated genes, indicating extensive secondary gene regulation in the deletion strain. Reduced gene expression levels can be caused by decreased expression or increased degradation. Whereas RNA sequencing cannot discern between these two events, miRNAs are well known to regulate mRNA stability and abundance [52]. To investigate whether mRNAs that are known targets of the upregulated miRNA let-7 family are downregulated, we constructed an miRNA–mRNA network using miRTarBase [31], where the connections between the upregulated group II let-7 miRNA family members let-7a, let-7b, let-7d, let-7f, let7-g, let-7i, and miR-98 and their predicted target mRNAs from miRTarBase are probed for miRNA–mRNA interaction. The miRNA–mRNA network shows that 94 of the 1933 potential let-7 family miRNA targets are downregulated in the TUT4/7 deletion strain (Figure 5, Table S10, Supplementary Files S12–S14). These data indicate that although the let-7 miRNAs are significantly more upregulated, only ~5% of their target mRNAs are downregulated. Whereas some mRNAs have binding sites for multiple let-7 miRNAs, miR-98 has the broadest substrate range, with 50 (28 unique) identified mRNA targets among the significantly downregulated mRNAs. Let-7b has 48 identified downregulated gene targets, including 23 unique targets, and let-7a has 32 downregulated targets, of which 11 are unique. On the other hand, let-7d, let-7f, let-7g, and let-7i share most of their targets; all 20 downregulated mRNA targets of let-7i are shared targets of the group II let-7 miRNAs.

These data confirm that while the deletion of TUT4/7 alters gene expression levels directly, the indirect regulation of gene expression levels via miRNAs is equally noteworthy. Thus, TUT4/7 are global contributors to both up- and downregulation of gene expression levels. In contrast, when we tried to construct a similar network for significantly downregulated miRNAs (the miR-296 family, miR-34 family, and miR200 family) and all upregulated (at least 2-fold) mRNAs, we found no notable miRNA–target interaction based on the database from miRTarBase v8.0. This further indicates that upregulated mRNAs are likely direct targets of TUT4/7 rather than targets of the downregulated miRNAs.

### 3.4. TUT4/7 Deletion Directly and Indirectly Regulates Protein Phosphorylation and Signaling

We previously showed that the let-7 family miRNAs, let-7a, let-7b, and let-7g, alter signalling by the oncogenic protein kinase AKT1 [27]. AKT1 is activated by phosphorylation at two regulatory sites, S473 and T308, and let-7 miRNA supplementation modulates AKT1 T308 and S473 phosphorylation in stimulated cells in a miRNA-specific manner [27]. We, therefore, probed AKT activation in TUT4/7 deleted cells, compared to WT cells. AKT is a major regulator of cellular signalling and cell differentiation. Interestingly, our sequencing data show a significant but small increase in the abundance of mRNAs with a role in regulation of protein phosphorylation, as well as regulation of AKT-interacting proteins in the STRING network of downregulated mRNAs (Figure 4A, Table S7), where 110 mRNAs with functions in protein phosphorylation are overabundant in the TUT4/7 deletion cell line (false discovery rate = 0.0286, *p*-value = 0.000223). Proteins in this pathway regulate cellular signalling pathways and are often involved in oncogenesis. We previously showed that let-7 miRNA family members regulate AKT1 phosphorylation, but not abundance, via the upstream pathway members, RICTOR and PIK3C2A [27].

#### 3.4.1. TUT4/7 Deletion Reduces AKT1 Levels

**Figure 6:**
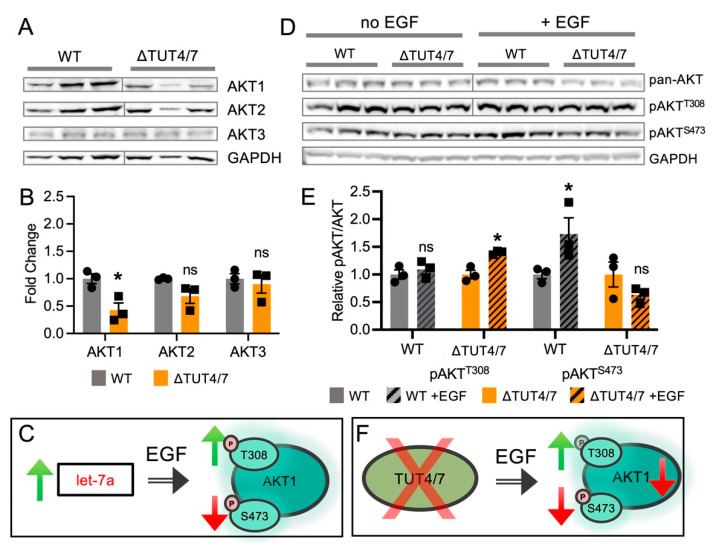
Messenger RNA and protein abundance are not directly related in the TUT4/7 deletion. (**A**) Western blots and (**B**) quantification of AKT isozymes in wild-type (black, circles) and ΔTUT4/7 (orange, squares) HEK 293T cells. (**C**) Artificial increase in let-7a levels by transfection was shown previously to increase T308 phosphorylation and reduce S473 phosphorylation of AKT1 in response to epidermal growth factor stimulation. (**D**) Western blots and (**E**) quantification of pan-AKT phosphorylation in wild-type (black, circles) and ΔTUT4/7 (orange, squares) HEK 293T cells. (**F**) Deletion of TUT4/7 alters response to EGF stimulation, increasing phosphorylation of AKT at T308 and reducing the response at S473. AKT1 specifically is decreased in ΔTUT4/7 cells. Blots were cropped to remove empty lanes (**A**) or molecular weight guides (**D**) for clarity. Asterisks indicate significance in Students *t*-test or ANOVA in GraphPad Prism where ns = not significant; * *p* < 0.05.

To investigate whether the observed let-7 miRNA increases in ΔTUT4/7 cells alter AKT1 phosphorylation and activity, we probed ΔTUT4/7 cells for AKT1 abundance and phosphorylation. First, we separately probed the abundance of the three endogenous AKT isozymes, AKT1, AKT2, and AKT3, with isozyme-specific antibodies. Western blot analysis revealed a significant 2.5-fold decrease in AKT1 abundance in ΔTUT4/7 cells, while AKT2 and AKT3 levels were not significantly changed (Figure 6A,B). As noted above, we have previously found a link between let-7 miRNAs, targets of TUT4/7, and AKT1 phosphorylation levels [27], guiding us to probe this change in AKT1 abundance further.

#### 3.4.2. Epidermal Growth Factor Stimulation and TUT4/7 Promote AKT Phosphorylation

AKT phosphorylation and activity is stimulated by epidermal growth factor (EGF) [53]. We previously showed EGF-dependent regulation of AKT1 phosphorylation by let-7 miRNAs; in the presence of EGF, let-7a drastically increased T308 phosphorylation, compared to EGF alone, while S473 phosphorylation is supressed by let-7a (Figure 6C) [27]. Let-7b and let-7g similarly suppressed S473 phosphorylation in response to EGF but had no effect on T308 phosphorylation [27]. Since let-7 family miRNAs are increased in abundance in the TUT4/7 deletion cells, we compared AKT abundance and phosphorylation levels in WT and ΔTUT4/7 cells with and without EGF stimulation, to test whether ΔTUT4/7 cells, which have increased let-7 levels, also show altered AKT phosphorylation levels. HEK 293T cells are known to constitutively activate AKT1 unless subjected to serum starvation [54]. As we previously showed for let-7a transfected cells, we now show that the phosphorylation of endogenous AKT in response to EGF stimulation is altered in ΔTUT4/7 cells. In cells lacking TUT4/7, the relative T308 phosphorylation increased significantly by 1.4-fold following EGF stimulation, while S473 phosphorylation showed no change (Figure 6D,E). The data indicate that in high-serum media, T308 is highly phosphorylated in wild-type cells, yet in ΔTUT4/7 deletion cells, EGF stimulation leads to a significant hyper-phosphorylation of T308. Conversely, while wild-type cells showed significant EGF-dependent S473 phosphorylation, EGF was unable to promote phosphorylation at S473 in cells lacking TUT4/7. The data suggest TUT4/7 deletion deregulated the signaling pathways upstream of T308 and S473 phosphorylation, as shown before for let-7a [27].

## 4. Discussion

The regulation of RNA homeostasis has long been known to be a prerequisite for controlling cellular metabolism, proliferation, and differentiation, but uridylation-dependent RNA decay remained a largely unexplored RNA decay pathway. The first description of RNA uridylation by the non-canonical poly (A) polymerase CID1 in *S. pombe*, now reclassed as a uridylyltransferase and member of the uridylyltransferase family, was published in 2007 [2], beginning a flurry of publications. Since then, uridylyltransferase homologs have been identified and characterized in several eukaryotes, including humans [5,55]. Uridylation-dependent decay has been shown to control the abundance of many different RNA species, including histone RNA [5], maternal mRNA [14,15], and both pre-miRNAs and mature miRNAs [10,21]. In humans, TUT4/7 are known to regulate multiple pathways, including histone mRNA degradation during cell cycle turnover [55], global transcriptome turnover of maternal mRNAs [15], miRNA degradation via polyuridylation [18,20], and group II pre-miRNA maturation via monouridylation [21]. Here, we captured the scope of cellular RNA uridylation by TUT4/7 and outline their impact on miRNA and mRNA homeostasis, cell proliferation, and the AKT1 oncogenic signaling pathway.

### 4.1. TUT4/7 Regulate Global RNA Turnover

Previously, RNA uridylation was thought to be triggered by specific cellular requirements, such as the degradation of maternal mRNA in a developing zygote [14] or let-7 miRNA degradation [10,22]. Our data show that RNA uridylation is a widespread, albeit non-essential mechanism for regulating RNA abundance. Our RNA sequencing data show that approximately 10% of mRNAs and 25% of miRNAs are significantly changed by at least 2-fold in a TUT4/7 knockout cell line, relative to wild type HEK 293T cells. This indicates that in human cells, uridylation is a major player in global RNA surveillance, rather than an RNA degradation pathway reserved for maternal mRNAs or specific miRNAs.

The impact of the TUT4/7 deletion on gene expression levels is comparable to CID1 deletion in *S. pombe*, where ~17% of mRNAs were differentially expressed [4]. In human cells, TUT4/7 regulated mRNA abundance both directly and indirectly. Whereas many mRNAs are decreased in abundance in the TUT4/7 deleted cells, an intriguing, almost 3-fold greater number of mRNAs increased in abundance. This deregulation could potentially be caused by reduced miRNA levels, where miRNAs fail to induce mRNA degradation. Similarly, reduced protein levels as a consequence of reduced gene expression of, for example, transcription factors, transcription repressors, or RNA-processing genes could lead to altered mRNA expression of downstream genes. Indeed, we found that RNA-processing genes are among the most deregulated genes in the deletion cells (Figure 3). Considering that a greater percentage of miRNAs are deregulated than mRNAs and that the deregulated mRNAs are largely targets of miRNA regulation, the major role of TUT4/7 may indeed be the regulation of miRNA metabolism.

It is not unusual that changes in transcript and protein levels do not align, yet it is particularly intriguing regarding the discrepancy between mRNA and protein abundance of the heat shock proteins. A major disruption and rearrangement of the transcriptome, and possibly proteome, would warrant increased expression of heat shock proteins to compensate for aberrantly produced proteins. This is, however, not the case in TUT4/7 deleted cells, as we did not observe an increase in heat shock proteins despite increased mRNA abundance. Therefore, we deduce that increased RNA abundance might be a direct effect of the TUT4/7 deletions, that is, failure to degrade the RNA. The protein production is then regulated at the mRNA level, leading to unchanged protein abundance. How translation of heat shock proteins in these cells is regulated remains to be elucidated.

Although many miRNAs were altered in abundance, we found family-specific clustering of up- and downregulated miRNAs, with the let-7 family of miRNAs showing the largest relative increases in the deletion strain. Further, we showed that many let-7 targets are downregulated through an miRNA–mRNA regulatory network. A previous study noted that the regulation of let-7 miRNAs and their mRNA targets is dependent on cancer type. In DU145 and IGROV-1 cells, miR-200c-3p and miR-141-3p are regulated in a cell-type-specific manner [17], whereas both miRNAs remained unchanged in abundance in HEK 293T cells, further corroborating the idea that TUT4/7-dependent regulation of miRNAs is cell-type specific.

A recent study showed 3p modifications in 27%–29% of miRNAs and monouridylation of miRNAs decreased from 15–17% to 2–5% upon TUT4/7 deletion [26]. In most cases, monouridylation was replaced by monoadenylation without altering miRNA abundance [26]. We found that group II let-7 miRNAs that were thought to require TUT4/7 monouridylation for DICER processing are not decreased but rather increased in abundance. It is likely that other nucleotidyltransferases can compensate for the TUT4/7 deletion. Indeed, the adenylyltransferase TENT2 (GLD2, PAPD4) has been shown to adenylate miRNAs in vitro [25,45] and may add the prerequisite nucleotide to group II miRNAs required for maturation.

In contrast, those miRNAs with decreased expression upon TUT4/7 deletion may be subject to more complex regulation mechanisms. For example, the simplest explanation would be that these miRNAs are direct targets of TUT4/7 and require monouridylation activity for maturation. However, this mechanism only applies to group II miRNAs, as group I miRNAs are processed by DICER and DROSHA directly from the pre-miRNA, without 3′ uridylation. Thus, a secondary mechanism of regulation is more likely to cause the reduced abundance of miRNAs upon TUT4/7 deletion. One possible pathway is through DICER directly; previous research shows global reduction in miRNA expression upon DICER knockout [56], and DICER is a target of the largely upregulated let-7 miRNAs. Therefore, the downregulated miRNAs found in this study may be a result of DICER downregulation or other similar mechanisms involving transcription factors or proteins involved in pre-miRNA transcription and processing.

### 4.2. TUT4/7 Regulate the miRNA–mRNA Network

The mapping of the miRNA–mRNA network showed that upregulated miRNAs interact with downregulated mRNAs, indicating that changes in gene expression levels can be attributed to altered miRNA abundance. Nonetheless, miRNA-mediated repression of mRNA does not always lead to mRNA degradation and may not be reflected on the gene expression level. In addition, while we show that TUT4/7 monouridylation of miRNAs is not required for maturation of group II miRNAs, monouridylation was previously shown to lead to tail-U-mediated repression (TUMR), modulating target recognition of miRNAs [26,35]. TUMR, thus, further complicates the miRNA–mRNA regulatory network. It is, therefore, not surprising that changes in mRNA levels are not always reflected on the protein level. In HEK 293T cells, the mRNA levels of heat shock proteins, HSP90AA1, HSP90AB1, HSP70, and HSP60, are significantly increased, yet protein levels of HSP90 and HSP70 remain unchanged. Interestingly, several miRNAs are known to regulate HSP90 expression. A recently identified substrate of TUT4/7 [26], known as miRNA-27a, downregulates the expression of HSP90 in esophageal squamous cell carcinoma [57]. Another study showed that miR-27a transfection led to decreased HSP90AA1 gene expression levels in human oral squamous cell carcinoma (HSC-4) cells under stress conditions but not under normal growth conditions [58]. Ourselves and others [26] showed that miR-27a abundance is unchanged in TUT4/7 deletion cells. Nonetheless, TUT4/7 deletion leads to a reduced uridylation of miR-27a [26], which impacts miRNA specificity, and can alter HSP90 expression. This corroborates that protein production is an intricately regulated network and cannot be simply deduced from transcriptomic data alone.

### 4.3. TUT4/7 Deletion Disrupts Cell Proliferation and Adhesion

The deletion of TUT4/7 leads to reduced cell proliferation, as observed here and previously [17]. A different study showed no change in doubling time upon TUT4/7 deletion but an approximately 1.5-fold increased doubling time when TUT4/7 were knocked out in combination with TENT2 deletion [35]. We additionally found that TUT4/7 deletion leads to the loss of cellular adhesion, along with the formation of multicellular aggregates and spheroid-like structures. Indeed, we previously noted that spheroid formation in epithelial ovarian cancer (EOC) cells, a critical step in EOC metastasis, correlates with high let-7 miRNA levels [27]. In the same study, we showed that let-7 miRNAs regulate the phosphorylation and activity of the oncogenic kinase AKT1 [27], a key regulator of cell proliferation. Indeed, we found that TUT4/7 deletion, which increases let-7 miRNA levels, deregulated AKT phosphorylation at T308 and S473, indicating a new role for TUT4/7 metabolism in regulating the PI3K/AKT pathway. AKT1 phosphorylation is likely regulated via let-7a directly, whereas TUT4/7 control AKT1, but not AKT2 or AKT3 protein abundance in a let-7a independent manner. Let-7a supplementation alone was previously shown to have no effect on AKT1 abundance [27]. Similarly, a recent study on HEK 293T cells showed that the deletion of DIS3L2 also results in increased phosphorylation of AKT at T308 [59]. Although TUT4/7 were previously suggested as chemotherapeutic targets, perturbation of uridylation-dependent RNA decay leads to potentially carcinogenic, increased AKT phosphorylation. Together, these data show the global impact of TUT4/7 on the human transcriptome and demonstrate that RNA uridylation is a general, rather than specialized, RNA degradation pathway.

## Data Availability

The datasets generated during this study are available at NCBI’s Gene Expression Omnibus and are accessible through GEO Series accession number GSE214610.

## Supplementary Materials

The following supporting information can be downloaded at: https://www.mdpi.com/article/10.3390/cells11233742/s1, Table S1–S10 and Supplementary files S1–S14. Table S1: Oligonucleotides used in this study; Table S2: miRNAs significantly increased in abundance > 2-fold in ∆TUT4/7; Table S3: miRNAs significantly decreased in abundance > 2-fold in ∆TUT4/7; Table S4: Differentially expressed miRNA genes (≥2-fold) raw counts; Table S5: miRNA families increased in abundance in ∆TUT4/7 cells. Individual miRNA family members are significantly increased by at least 2-fold; Table S6: miRNA families decreased in abundance in ∆TUT4/7 cells. Individual miRNA family members are significantly increased by at least 2-fold; Table S7: Group II miRNA decreased or increased by at least 2-fold in abundance, or unchanged in ∆TUT4/7 cells compared to WT cells; Table S8: Gene Ontology (GO)—biological process analysis of mRNAs increased in abundance in ∆TUT4/7 cells; Table S9: Gene Ontology (GO)—biological process analysis of mRNAs decreased in abundance in ∆TUT4/7 cells; Table S10: Target mapping in the miRNA/mRNA network. DE = significantly differentially expressed by at least 2-fold. Parentheses indicate number of target genes out of total number of target genes that are decreased in abundance in ∆TUT4/7 cells. Shared targets include mRNA targeted by multiple let-7 family miRNAs.
