# Supplementary material for: Terminal Uridylyltransferases TUT4/7 Regulate microRNA and mRNA Homeostasis
Source: Cells. 2022 Nov 23;11(23):3742. doi: 10.3390/cells11233742 (PMC9736393; doi:10.3390/cells11233742)
Supplement: Supplementary file 1 [file cells-11-03742-s001.zip › cells-2007303-supplementary.pdf]

Terminal Uridylyltransferases TUT4/7 Regulate microRNA and mRNA Homeostasis

Pengcheng Zhang †, Mallory I. Frederick † and Ilka U. Heinemann *

Department of Biochemistry, the University of Western Ontario, 1151 Richmond Street, London, ON N6A 5C1, Canada
* Correspondence: ilka.heinemann@uwo.ca
† These authors contributed equally to this work.

Supplementary Material:
Supplementary Tables S1-S10

Supplementary Table S1: Oligonucleotides used in this study

Name	Oligonucleotide (5′-3′)
hsa-let-7a-5p, RT-PCR	GTCGTATGCAGAGCAGGGTCCGAGGTATTCGCACTGC ATACGACAACTAT
hsa-let-7b-5p, RT-PCR	GTCGTATGCAGAGCAGGGTCCGAGGTATTCGCACTGC ATACGACAACCAC
hsa-let-7c-5p, RT-PCR	GTCGTATGCAGAGCAGGGTCCGAGGTATTCGCACTGC ATACGACAACCAT
hsa-let-7d-5p, RT-PCR	GTCGTATGCAGAGCAGGGTCCGAGGTATTCGCACTGC ATACGACAACTAT
hsa-let-7e-5p, RT-PCR	GTCGTATGCAGAGCAGGGTCCGAGGTATTCGCACTGC ATACGACAACTAT
hsa-let-7f-5p, RT-PCR	GTCGTATGCAGAGCAGGGTCCGAGGTATTCGCACTGC ATACGACAACTAT
hsa-let-7g-5p, RT-PCR	GTCGTATGCAGAGCAGGGTCCGAGGTATTCGCACTGC ATACGACAACTGT
hsa-let-7i-5p, RT-PCR	GTCGTATGCAGAGCAGGGTCCGAGGTATTCGCACTGC ATACGACAACAGC
hsa-mir-98-5p, RT-PCR	GTCGTATGCAGAGCAGGGTCCGAGGTATTCGCACTGC ATACGACAACAAT
SNORD47, RT-PCR	GTCGTATGCAGAGCAGGGTCCGAGGTATTCGCACTGC ATACGACAACCTC
hsa-let-7a-5p, qPCR, forward	AGGCTGAGGTAGTAGGTTG
hsa-let-7b-5p, qPCR, forward	AGGCTGAGGTAGTAGGTTG
hsa-let-7c-5p, qPCR, forward	AGGCTGAGGTAGTAGGTTG
hsa-let-7d-5p, qPCR, forward	AGGCAGAGGTAGTAGGTTG
hsa-let-7e-5p, qPCR, forward	AGGCTGAGGTAGGAGGTTG

hsa-let-7f-5p, qPCR, forward	AGGCTGAGGTAGTAGATTG
hsa-let-7g-5p, qPCR, forward	AGGCTGAGGTAGTAGTTTG
hsa-let-7i-5p, qPCR, forward	AGGCTGAGGTAGTAGTTTG
hsa-mir-98-5p, qPCR, forward	AGGCTGAGGTAGTAAGTTG
SNORD47, qPCR, forward	ATCACTGTAAAACCGTTCCA
universal (for miRNA), qPCR, reverse	GAGCAGGGTCCGAGGT
RPS18, qPCR, forward	CAACACCAACATCGATGGGC
RPS18, qPCR, reverse	GGTGATCACACGTTCCACCT
HSPA8, qPCR, forward	GCTGCTGCTATTGCTTACGG
HSPA8, qPCR, reverse	GCTGGAAGAGAGGGTACGC
HSP90AA1, qPCR, forward	AGTGCTCGAGTCACATTCTGC
HSP90AA1, qPCR, reverse	CAACCCTTGGAGCAGCTAGTG
HSP90AB1, qPCR, forward	TATGTCCGCCGTGTGTTCAT
HSP90AB1, qPCR, reverse	TCTGCTGGAGCATTTCTCGG
HSPD1, qPCR, forward	CCACAGTCTTTCGCCAGATGA
HSPD1, qPCR, reverse	CACAGCATCGGCTAAAAGGTC

Supplementary Table S2: miRNAs significantly increased in abundance > 2-fold in ΔTUT4/7.

microRNA ID	Fold Change	P-value	False Discovery Rate
hsa-miR-504-5p	34.00	1.74E-10	1.20E-08
hsa-let-7i-3p	32.60	9.85E-11	8.97E-09
hsa-let-7i-5p	31.40	1.13E-14	9.36E-12
hsa-let-7d-3p	24.20	1.35E-09	7.45E-08
hsa-miR-887-3p	23.20	6.21E-10	3.94E-08
hsa-let-7d-5p	19.20	2.34E-12	3.86E-10
hsa-let-7f-1-3p	16.00	1.20E-09	7.07E-08
hsa-miR-651-5p	15.40	6.88E-09	2.99E-07
hsa-miR-5682	12.60	2.57E-09	1.18E-07
hsa-let-7b-5p	11.60	2.18E-12	3.86E-10
hsa-let-7b-3p	11.10	3.19E-08	1.00E-06
hsa-miR-6765-3p	8.13	8.11E-08	2.16E-06
hsa-miR-98-3p	6.59	1.62E-07	3.93E-06
hsa-let-7g-3p	6.42	9.16E-07	1.54E-05
hsa-let-7g-5p	6.18	1.60E-09	8.26E-08
hsa-miR-4783-3p	5.54	6.02E-07	1.13E-05
hsa-miR-3168	5.35	1.59E-03	5.71E-03
hsa-miR-7-5p	4.87	5.57E-08	1.53E-06
hsa-miR-98-5p	4.79	2.67E-08	8.83E-07
hsa-miR-4326	4.74	3.18E-07	6.56E-06
hsa-miR-4428	4.73	5.86E-06	7.56E-05

hsa-miR-129-5p	4.65	2.82E-07	6.25E-06
hsa-miR-1229-3p	4.56	2.81E-04	1.52E-03
hsa-miR-129-1-3p	4.22	6.65E-06	8.45E-05
hsa-miR-3171	4.10	2.44E-06	3.42E-05
hsa-miR-6850-5p	3.95	2.36E-04	1.35E-03
hsa-miR-18a-3p	3.87	5.67E-06	7.43E-05
hsa-miR-3176	3.73	6.82E-07	1.24E-05
hsa-miR-129-2-3p	3.64	7.76E-06	9.57E-05
hsa-miR-19a-5p	3.64	7.17E-05	5.60E-04
hsa-miR-636	3.64	4.62E-04	2.17E-03
hsa-miR-3615	3.46	7.75E-05	5.73E-04
hsa-miR-3620-3p	3.44	6.60E-05	5.28E-04
hsa-miR-6858-5p	3.33	3.06E-04	1.58E-03
hsa-miR-3144-3p	3.30	1.30E-06	2.03E-05
hsa-miR-891a-5p	3.19	4.71E-05	4.14E-04
hsa-let-7a-3p	3.12	4.96E-07	9.75E-06
hsa-miR-1266-5p	3.09	1.51E-05	1.54E-04
hsa-miR-6807-5p	3.07	4.47E-05	4.00E-04
hsa-miR-7108-5p	3.05	3.79E-04	1.87E-03
hsa-miR-301a-5p	3.03	1.52E-05	1.54E-04
hsa-miR-4435	2.98	7.91E-05	5.73E-04
hsa-miR-873-3p	2.91	1.09E-04	7.57E-04
hsa-miR-6768-5p	2.89	1.86E-04	1.12E-03
hsa-miR-208a-3p	2.87	1.17E-04	7.96E-04
hsa-miR-3182	2.84	1.21E-05	1.31E-04
hsa-miR-1914-3p	2.82	1.44E-03	5.38E-03
hsa-miR-452-5p	2.82	1.34E-05	1.40E-04
hsa-let-7f-2-3p	2.79	8.58E-06	1.04E-04
hsa-miR-760	2.78	6.65E-05	5.28E-04
hsa-miR-320a-5p	2.71	5.81E-04	2.60E-03
hsa-miR-573	2.71	1.58E-04	1.01E-03
hsa-miR-19b-1-5p	2.68	9.82E-06	1.13E-04
hsa-miR-134-5p	2.67	1.58E-04	1.01E-03
hsa-let-7f-5p	2.62	1.47E-08	5.38E-07
hsa-miR-654-3p	2.58	2.83E-04	1.52E-03
hsa-miR-664b-5p	2.56	1.51E-04	9.76E-04
hsa-miR-29b-2-5p	2.55	2.35E-05	2.28E-04
hsa-miR-3187-3p	2.54	2.72E-04	1.49E-03
hsa-miR-873-5p	2.52	2.02E-04	1.17E-03
hsa-miR-101-2-5p	2.51	1.27E-04	8.36E-04

hsa-miR-195-3p	2.50	1.03E-05	1.16E-04
hsa-miR-1247-5p	2.47	2.72E-03	8.67E-03
hsa-miR-3144-5p	2.47	1.60E-04	1.01E-03
hsa-miR-4470	2.46	1.92E-04	1.15E-03
hsa-miR-4775	2.45	5.33E-04	2.42E-03
hsa-miR-194-3p	2.43	3.46E-04	1.72E-03
hsa-miR-92a-1-5p	2.43	3.20E-04	1.63E-03
hsa-miR-103a-2-5p	2.42	1.86E-03	6.37E-03
hsa-miR-3679-5p	2.41	1.69E-04	1.06E-03
hsa-miR-579-5p	2.38	1.05E-04	7.35E-04
hsa-miR-320d	2.36	3.29E-04	1.66E-03
hsa-miR-1914-5p	2.33	1.27E-02	3.08E-02
hsa-miR-3131	2.32	5.96E-04	2.65E-03
hsa-miR-3928-3p	2.32	8.45E-05	6.02E-04
hsa-miR-3143	2.29	8.43E-05	6.02E-04
hsa-miR-6742-5p	2.29	6.70E-04	2.91E-03
hsa-miR-6821-5p	2.28	2.86E-03	9.00E-03
hsa-miR-941	2.28	1.53E-05	1.54E-04
hsa-miR-1226-3p	2.24	1.22E-04	8.21E-04
hsa-miR-4791	2.23	7.67E-04	3.28E-03
hsa-miR-1236-5p	2.22	3.31E-03	1.02E-02
hsa-miR-381-3p	2.22	1.57E-03	5.70E-03
hsa-miR-379-5p	2.21	7.86E-04	3.33E-03
hsa-miR-92a-3p	2.21	6.50E-05	5.28E-04
hsa-miR-19b-2-5p	2.20	1.21E-03	4.66E-03
hsa-miR-3940-3p	2.19	1.78E-03	6.18E-03
hsa-miR-2355-5p	2.18	5.79E-04	2.60E-03
hsa-miR-10396a-3p	2.17	1.67E-03	5.99E-03
hsa-miR-3616-3p	2.17	2.09E-03	6.97E-03
hsa-miR-382-5p	2.17	2.11E-03	7.00E-03
hsa-miR-1180-3p	2.14	2.02E-04	1.17E-03
hsa-miR-4725-3p	2.13	3.88E-03	1.17E-02
hsa-miR-320a-3p	2.12	4.45E-04	2.11E-03
hsa-miR-365a-5p	2.12	2.20E-03	7.22E-03
hsa-miR-503-5p	2.12	1.96E-06	2.78E-05
hsa-miR-494-3p	2.11	1.02E-02	2.59E-02
hsa-miR-2276-3p	2.10	4.14E-04	2.02E-03
hsa-miR-503-3p	2.09	4.50E-05	4.00E-04
hsa-miR-181d-5p	2.08	7.18E-05	5.60E-04
hsa-miR-200c-5p	2.08	1.90E-03	6.48E-03

hsa-miR-3162-5p	2.08	1.50E-03	5.50E-03
hsa-miR-6800-3p	2.08	1.29E-03	4.95E-03
hsa-miR-671-5p	2.06	5.33E-06	7.10E-05
hsa-miR-10399-5p	2.05	2.69E-02	5.50E-02
hsa-miR-20a-5p	2.05	1.13E-06	1.79E-05
hsa-miR-3911	2.05	1.48E-03	5.45E-03
hsa-miR-484	2.05	9.64E-04	3.91E-03
hsa-miR-1257	2.04	1.15E-03	4.47E-03
hsa-miR-3187-5p	2.04	2.53E-03	8.19E-03
hsa-miR-6892-5p	2.04	1.14E-02	2.83E-02
hsa-miR-1180-5p	2.03	1.96E-03	6.64E-03
hsa-miR-141-5p	2.01	1.58E-03	5.70E-03
hsa-miR-20a-3p	2.01	9.57E-06	1.11E-04
hsa-miR-888-5p	2.01	4.01E-03	1.19E-02

Supplementary Table S3: miRNAs significantly decreased in abundance > 2-fold in ΔTUT4/7.

microRNA ID	Fold Change	P-value	False Discovery Rate
hsa-miR-296-5p	-142.00	1.57E-13	6.05E-11
hsa-miR-296-3p	-136.00	2.20E-13	6.05E-11
hsa-miR-34c-5p	-63.20	4.83E-12	6.65E-10
hsa-miR-196a-1-3p	-38.30	4.04E-11	4.76E-09
hsa-miR-34b-5p	-31.60	1.09E-10	8.97E-09
hsa-miR-34b-3p	-23.30	1.71E-10	1.20E-08
hsa-miR-34c-3p	-13.50	2.10E-09	1.02E-07
hsa-miR-489-3p	-12.40	2.96E-07	6.26E-06
hsa-miR-874-3p	-10.00	1.50E-08	5.38E-07
hsa-miR-4778-3p	-8.87	1.35E-08	5.32E-07
hsa-miR-653-5p	-7.79	3.27E-08	1.00E-06
hsa-miR-653-3p	-7.49	2.04E-07	4.67E-06
hsa-miR-767-5p	-7.07	7.08E-07	1.24E-05
hsa-miR-200b-3p	-6.72	1.03E-06	1.67E-05
hsa-miR-200a-3p	-6.51	1.05E-07	2.71E-06
hsa-miR-4664-3p	-6.20	2.87E-07	6.25E-06
hsa-miR-1468-5p	-5.92	4.77E-08	1.36E-06
hsa-miR-204-5p	-5.87	1.82E-08	6.28E-07
hsa-miR-152-3p	-5.34	8.39E-09	3.46E-07
hsa-miR-34a-3p	-5.17	3.67E-08	1.08E-06
hsa-miR-34a-5p	-4.76	7.86E-11	8.11E-09
hsa-miR-4421	-4.73	3.48E-06	4.78E-05

hsa-miR-216a-5p	-4.53	1.44E-06	2.21E-05
hsa-miR-429	-4.48	1.78E-06	2.62E-05
hsa-miR-143-3p	-4.23	8.72E-06	1.04E-04
hsa-miR-4443	-4.08	1.83E-06	2.65E-05
hsa-miR-215-3p	-3.99	6.02E-05	5.11E-04
hsa-miR-874-5p	-3.90	7.49E-06	9.37E-05
hsa-miR-301b-3p	-3.56	9.07E-06	1.07E-04
hsa-miR-4664-5p	-3.40	1.68E-05	1.67E-04
hsa-miR-187-3p	-3.37	1.08E-07	2.71E-06
hsa-miR-556-5p	-3.27	3.16E-04	1.62E-03
hsa-miR-4778-5p	-3.11	7.91E-05	5.73E-04
hsa-miR-217-5p	-3.05	9.78E-04	3.94E-03
hsa-miR-31-3p	-3.04	3.79E-05	3.48E-04
hsa-miR-105-5p	-3.02	1.81E-04	1.11E-03
hsa-miR-211-5p	-3.00	5.37E-05	4.62E-04
hsa-miR-3145-3p	-2.94	9.07E-04	3.75E-03
hsa-miR-551b-3p	-2.87	1.06E-05	1.19E-04
hsa-miR-125b-5p	-2.86	1.28E-04	8.38E-04
hsa-miR-6505-3p	-2.83	7.91E-05	5.73E-04
hsa-miR-6514-5p	-2.77	7.46E-05	5.71E-04
hsa-miR-577	-2.75	2.77E-05	2.66E-04
hsa-miR-499a-5p	-2.72	1.96E-04	1.15E-03
hsa-miR-210-3p	-2.69	5.33E-07	1.02E-05
hsa-miR-1179	-2.64	1.22E-04	8.21E-04
hsa-miR-30a-3p	-2.61	1.58E-06	2.37E-05
hsa-miR-24-3p	-2.58	6.92E-07	1.24E-05
hsa-miR-2114-5p	-2.57	2.88E-03	9.03E-03
hsa-miR-181a-2-3p	-2.48	5.19E-06	7.03E-05
hsa-miR-31-5p	-2.47	2.04E-07	4.67E-06
hsa-miR-6780b-3p	-2.44	2.68E-04	1.48E-03
hsa-miR-4665-5p	-2.43	2.39E-04	1.36E-03
hsa-miR-4423-5p	-2.42	2.83E-04	1.52E-03
hsa-miR-148a-3p	-2.38	4.47E-04	2.11E-03
hsa-miR-641	-2.38	1.34E-05	1.40E-04
hsa-miR-340-3p	-2.36	1.06E-02	2.67E-02
hsa-miR-26b-3p	-2.35	4.20E-04	2.03E-03
hsa-miR-23a-3p	-2.33	1.25E-04	8.29E-04
hsa-miR-1295a	-2.32	1.83E-04	1.12E-03
hsa-miR-488-3p	-2.32	9.83E-04	3.94E-03
hsa-miR-99a-5p	-2.29	4.84E-05	4.21E-04

hsa-miR-196a-5p	-2.24	3.63E-07	7.31E-06
hsa-miR-30c-5p	-2.24	5.08E-04	2.35E-03
hsa-miR-99a-3p	-2.22	1.41E-04	9.16E-04
hsa-miR-542-3p	-2.21	7.31E-05	5.64E-04
hsa-miR-6505-5p	-2.20	2.90E-03	9.05E-03
hsa-miR-664a-3p	-2.20	6.60E-03	1.79E-02
hsa-miR-28-5p	-2.19	2.68E-04	1.48E-03
hsa-miR-542-5p	-2.19	6.18E-05	5.16E-04
hsa-miR-374c-5p	-2.18	4.42E-03	1.29E-02
hsa-miR-1843	-2.17	6.49E-05	5.28E-04
hsa-miR-23b-3p	-2.17	8.27E-07	1.42E-05
hsa-miR-26b-5p	-2.17	9.39E-07	1.55E-05
hsa-miR-548l	-2.13	4.10E-04	2.02E-03
hsa-miR-2113	-2.12	4.09E-03	1.21E-02
hsa-miR-218-2-3p	-2.10	1.27E-02	3.07E-02
hsa-miR-3175	-2.09	1.13E-03	4.43E-03
hsa-miR-3179	-2.09	1.70E-03	6.02E-03
hsa-miR-5187-5p	-2.09	8.64E-04	3.61E-03
hsa-miR-450a-5p	-2.08	5.83E-04	2.60E-03
hsa-miR-4735-5p	-2.07	1.42E-03	5.33E-03
hsa-miR-1227-3p	-2.06	4.30E-02	8.07E-02
hsa-miR-6726-3p	-2.06	3.59E-03	1.09E-02
hsa-miR-3660	-2.05	2.35E-03	7.65E-03
hsa-miR-6791-3p	-2.05	5.95E-03	1.66E-02
hsa-miR-142-3p	-2.04	1.31E-03	4.99E-03
hsa-miR-664a-5p	-2.04	7.15E-04	3.08E-03
hsa-miR-450a-2-3p	-2.02	8.14E-04	3.43E-03
hsa-miR-7112-3p	-2.02	2.90E-03	9.05E-03
hsa-miR-140-5p	-2.01	4.32E-03	1.27E-02

Supplementary Table S4: Differentially expressed miRNA genes (>= 2-fold) raw counts

microRNA I	D1	D2	D3	W1	W2	W3
hsa-let-7a-3p	945	803	712	207	459	239
hsa-let-7b-3p	101647	96526	98960	53099	101341	58495
hsa-let-7b-5p	157	131	143	3	16	11
hsa-let-7d-3p	51695	53076	54879	3907	7325	4459
hsa-let-7d-5p	149	159	124	146	284	155
hsa-let-7f-1-3p	100678	95106	97087	53438	101227	58526
hsa-let-7f-2-3p	520	726	987	19	59	20
hsa-let-7f-5p	27337	28039	28192	1285	1977	1529
hsa-let-7g-3p	117	101	84	95	153	95
hsa-let-7g-5p	31257	30049	30461	33354	61718	34482
hsa-let-7i-3p	100	119	99	1	3	3
hsa-let-7i-5p	274	223	187	68	115	80
hsa-miR-101-2-5p	252758	224727	213966	75923	142356	83484
hsa-miR-10396a-3p	191	244	281	24	34	47
hsa-miR-10399-5p	132913	125400	121599	15470	28528	24092
hsa-miR-103a-2-5p	785	800	925	15	25	28
hsa-miR-105-5p	287806	259899	249705	7349	13325	8169
hsa-miR-1179	14	18	14	28	62	28
hsa-miR-1180-3p	116	128	120	31	61	58
hsa-miR-1180-5p	63	68	72	56	73	38
hsa-miR-1226-3p	53	65	77	31	30	42
hsa-miR-1227-3p	9	22	20	27	44	39
hsa-miR-1229-3p	12	13	8	2	1	4
hsa-miR-1236-5p	33	30	36	15	5	23
hsa-miR-1247-5p	3	3	2	0	0	2
hsa-miR-1257	3	1	11	0	0	0
hsa-miR-125b-5p	6	8	4	3	3	10
hsa-miR-1266-5p	31	36	27	19	8	4
hsa-miR-129-1-3p	293	264	318	238	374	131
hsa-miR-129-2-3p	5	4	9	2	0	3
hsa-miR-129-5p	4	3	7	1	0	0
hsa-miR-1295a	1	0	4	17	17	14
hsa-miR-134-5p	54	62	58	31	53	51
hsa-miR-140-5p	5	7	5	11	19	14
hsa-miR-141-5p	8	6	5	10	20	7
hsa-miR-142-3p	279	268	194	150	236	221
hsa-miR-143-3p	4970	5164	5265	5426	8961	4300

hsa-miR-1468-5p	3051	3183	2445	3280	5732	5907
hsa-miR-148a-3p	257	268	297	252	332	143
hsa-miR-152-3p	7981	7129	6133	5616	11420	6870
hsa-miR-181a-2-3p	126726	117116	116484	140844	213833	85949
hsa-miR-181d-5p	1555	1383	1230	1735	3328	1806
hsa-miR-1843	21386	19215	19036	40271	60212	24687
hsa-miR-187-3p	15	13	8	40	52	38
hsa-miR-18a-3p	3101	3579	4220	2007	2734	1107
hsa-miR-1914-3p	32	47	49	14	30	20
hsa-miR-1914-5p	27	23	25	10	15	19
hsa-miR-194-3p	6	7	8	3	3	2
hsa-miR-195-3p	208	209	176	161	266	169
hsa-miR-196a-1-3p	4	1	2	5	14	7
hsa-miR-196a-5p	0	3	3	2	2	0
hsa-miR-19a-5p	13	19	15	8	17	8
hsa-miR-19b-1-5p	1530	1961	2470	930	1429	689
hsa-miR-19b-2-5p	16	13	15	7	13	5
hsa-miR-200a-3p	5	8	5	8	15	31
hsa-miR-200b-3p	4	5	7	6	8	2
hsa-miR-200c-5p	6	12	5	8	3	3
hsa-miR-204-5p	21	35	71	6	15	1
hsa-miR-208a-3p	4	2	8	1	2	0
hsa-miR-20a-3p	1	1	5	1	1	5
hsa-miR-20a-5p	5	8	12	0	0	3
hsa-miR-210-3p	6	10	25	23	30	9
hsa-miR-211-5p	8	4	10	19	19	4
hsa-miR-2113	10	7	8	3	6	6
hsa-miR-2114-5p	26	30	60	8	31	10
hsa-miR-215-3p	2942	2483	2719	1327	2235	1270
hsa-miR-216a-5p	27	45	61	31	35	10
hsa-miR-217-5p	3	4	4	0	3	2
hsa-miR-218-2-3p	110	99	79	48	78	49
hsa-miR-2276-3p	6	1	12	8	10	6
hsa-miR-2355-5p	37	32	48	25	45	20
hsa-miR-23a-3p	3	2	1	5	3	0
hsa-miR-23b-3p	79	73	55	21	48	37
hsa-miR-24-3p	366	387	411	363	585	482
hsa-miR-26b-3p	32531	36043	39934	64545	121272	52058
hsa-miR-26b-5p	98	93	86	164	233	95
hsa-miR-28-5p	28	13	30	75	139	56

hsa-miR-296-3p	19981	18804	17961	12348	25412	15678
hsa-miR-296-5p	5722	5134	4524	3939	7598	4724
hsa-miR-29b-2-5p	0	1	0	3	3	3
hsa-miR-301a-5p	178	147	126	303	352	285
hsa-miR-301b-3p	145	131	130	103	191	123
hsa-miR-30a-3p	4	4	7	0	3	0
hsa-miR-30c-5p	64	59	54	19	23	11
hsa-miR-31-3p	35	46	58	22	62	32
hsa-miR-31-5p	0	2	3	3	11	3
hsa-miR-3131	0	3	2	4	10	6
hsa-miR-3143	3	1	2	4	1	2
hsa-miR-3144-3p	1154	1178	1195	694	1383	601
hsa-miR-3144-5p	6	0	3	2	0	3
hsa-miR-3145-3p	12	14	13	12	22	15
hsa-miR-3162-5p	5	3	6	0	0	0
hsa-miR-3168	21	29	46	21	35	12
hsa-miR-3171	56	54	41	23	54	38
hsa-miR-3175	82	125	112	128	170	176
hsa-miR-3176	229	288	291	191	203	176
hsa-miR-3179	98	75	85	102	188	84
hsa-miR-3182	242	216	289	174	343	132
hsa-miR-3187-3p	20801	20136	19681	20099	39925	20770
hsa-miR-3187-5p	47	62	57	70	117	76
hsa-miR-320a-3p	21	30	23	10	22	14
hsa-miR-320a-5p	212	202	217	201	432	180
hsa-miR-320d	5	9	13	6	14	4
hsa-miR-340-3p	56	58	70	14	19	5
hsa-miR-34a-3p	47	57	58	9	14	14
hsa-miR-34a-5p	566	494	541	72	175	131
hsa-miR-34b-3p	604	402	375	271	716	207
hsa-miR-34b-5p	3	8	10	1	4	1
hsa-miR-34c-3p	114	137	192	80	238	102
hsa-miR-34c-5p	16	10	20	14	28	18
hsa-miR-3615	23	26	13	26	68	42
hsa-miR-3616-3p	69	73	90	207	307	128
hsa-miR-3620-3p	18	42	34	18	9	15
hsa-miR-365a-5p	3	13	8	2	5	1
hsa-miR-3660	88	159	189	100	139	57
hsa-miR-3679-5p	1	1	4	0	2	2
hsa-miR-374c-5p	53	44	31	14	42	29

hsa-miR-379-5p	141	123	92	104	203	165
hsa-miR-381-3p	29	28	27	57	79	35
hsa-miR-382-5p	189	300	586	322	803	248
hsa-miR-3911	4988	6024	8771	4416	6819	2693
hsa-miR-3928-3p	6624	6021	5694	5281	8272	8315
hsa-miR-3940-3p	4660	4139	3782	5944	11516	8023
hsa-miR-429	627	675	662	783	1379	810
hsa-miR-4326	2862	2727	2555	2808	4376	2469
hsa-miR-4421	506	484	381	244	399	168
hsa-miR-4423-5p	14	25	12	15	22	11
hsa-miR-4428	29	24	23	7	19	14
hsa-miR-4435	11	15	15	1	3	3
hsa-miR-4443	12	9	26	17	29	5
hsa-miR-4470	4	1	5	4	6	1
hsa-miR-450a-2-3p	3	4	2	1	0	0
hsa-miR-450a-5p	27	20	26	17	32	21
hsa-miR-452-5p	2	2	0	1	6	0
hsa-miR-4664-3p	10	8	11	2	8	16
hsa-miR-4664-5p	5	6	7	6	13	6
hsa-miR-4665-5p	339	300	307	203	316	146
hsa-miR-4725-3p	17	16	23	10	13	10
hsa-miR-4735-5p	50	32	41	26	61	74
hsa-miR-4775	6327	5725	5353	7924	16806	7551
hsa-miR-4778-3p	1992	1872	1504	2203	4513	5005
hsa-miR-4778-5p	360	398	340	431	692	453
hsa-miR-4783-3p	26	27	22	12	11	9
hsa-miR-4791	184	158	145	272	394	418
hsa-miR-484	7	9	7	14	22	25
hsa-miR-488-3p	14	12	4	50	88	57
hsa-miR-489-3p	1	6	3	2	0	2
hsa-miR-494-3p	6	4	6	3	8	3
hsa-miR-499a-5p	17	22	19	122	210	137
hsa-miR-503-3p	194	165	153	138	242	160
hsa-miR-503-5p	24	17	27	16	16	7
hsa-miR-504-5p	2544	2269	2096	1524	3192	1605
hsa-miR-5187-5p	43	41	48	57	83	48
hsa-miR-542-3p	9	7	4	11	23	13
hsa-miR-542-5p	92527	78407	67014	115547	241013	259518
hsa-miR-548l	297	275	323	286	307	149
hsa-miR-551b-3p	46829	43867	43946	46504	75747	56072

hsa-miR-556-5p	27	31	30	31	31	13
hsa-miR-5682	1975	2301	2574	2076	3216	1400
hsa-miR-573	11	21	27	15	42	17
hsa-miR-577	81	97	106	81	134	102
hsa-miR-579-5p	1	2	1	1	1	6
hsa-miR-636	0	3	3	1	1	3
hsa-miR-641	456	485	511	1913	4567	2624
hsa-miR-6505-3p	2	2	3	1	13	2
hsa-miR-6505-5p	204	187	201	145	187	162
hsa-miR-651-5p	18	28	16	26	47	39
hsa-miR-6514-5p	7	5	4	7	6	5
hsa-miR-653-3p	108	111	132	95	90	111
hsa-miR-653-5p	8011	7724	7331	11014	15474	11117
hsa-miR-654-3p	6936	6451	5735	5443	11152	7142
hsa-miR-664a-3p	25839	26241	24959	38559	72932	39271
hsa-miR-664a-5p	355	371	318	420	636	328
hsa-miR-664b-5p	376	352	313	296	501	302
hsa-miR-671-5p	897408	856917	818515	1060000	2230000	1020000
hsa-miR-6726-3p	5896	5818	5832	3453	4063	2250
hsa-miR-6742-5p	23785	21786	17122	10599	21859	34599
hsa-miR-6765-3p	283	256	234	657	1063	505
hsa-miR-6768-5p	742	712	602	645	1134	564
hsa-miR-6780b-3p	6	15	12	16	32	28
hsa-miR-6791-3p	10	11	11	19	38	23
hsa-miR-6800-3p	2	1	2	2	4	3
hsa-miR-6807-5p	3	7	2	10	5	4
hsa-miR-6821-5p	283	237	229	202	361	188
hsa-miR-6850-5p	80	72	70	64	70	36
hsa-miR-6858-5p	5	2	7	2	6	4
hsa-miR-6892-5p	351	351	295	163	233	134
hsa-miR-7-5p	67	46	60	71	92	104
hsa-miR-7108-5p	23374	20898	20110	25312	50514	28443
hsa-miR-7112-3p	139	118	153	193	270	199
hsa-miR-760	16834	15293	15404	19690	37521	21765
hsa-miR-767-5p	3	6	7	7	17	18
hsa-miR-873-3p	77	77	90	163	301	165
hsa-miR-873-5p	97	130	203	111	183	102
hsa-miR-874-3p	14449	14358	13623	12663	23509	10675
hsa-miR-874-5p	18	25	31	20	28	35
hsa-miR-887-3p	76786	68340	62087	41599	75079	42378

hsa-miR-888-5p	371	385	308	1148	1994	1017
hsa-miR-891a-5p	11	11	14	24	40	21
hsa-miR-92a-1-5p	16	16	17	22	41	40
hsa-miR-92a-3p	398	432	387	301	435	459
hsa-miR-941	2498	2646	2869	948	989	374
hsa-miR-98-3p	46729	44310	37636	21574	37285	40032
hsa-miR-98-5p	261	252	246	93	172	232
hsa-miR-99a-3p	15	31	29	36	49	40
hsa-miR-99a-5p	1	1	4	3	1	1

Supplementary Table S5: miRNA families increased in abundance in ΔTUT4/7 cells. Individual miRNA family members are significantly increased by at least 2-fold.

miRNA Family	DE-miRNA Count	miRNA Family Size	P-value	miRNA (significantly changed)
let-7	10	12	4.77E-11	hsa-let-7a-1,hsa-let-7a-2,hsa-let-7a-3,hsa-let-7b,hsa-let-7d,hsa-let-7f-1,hsa-let-7f-2,hsa-let-7g,hsa-let-7i,hsa-mir-98
mir-7	3	3	2.58E-04	hsa-mir-7-1,hsa-mir-7-2,hsa-mir-7-3
mir-129	2	2	4.08E-03	hsa-mir-129-1,hsa-mir-129-2
mir-19	3	3	2.58E-04	hsa-mir-19a,hsa-mir-19b-1,hsa-mir-19b-2
mir-4435	2	2	4.08E-03	hsa-mir-4435-1,hsa-mir-4435-2
mir-320	3	8	0.0114	hsa-mir-320a,hsa-mir-320d-1,hsa-mir-320d-2
mir-194	2	2	4.08E-03	hsa-mir-194-1,hsa-mir-194-2
mir-25	2	4	0.0225	hsa-mir-92a-1,hsa-mir-92a-2
mir-941	5	5	1.00E-06	hsa-mir-941-1,hsa-mir-941-2,hsa-mir-941-3,hsa-mir-941-4,hsa-mir-941-5
mir-8	2	5	0.0359	hsa-mir-141,hsa-mir-200c

Supplementary Table S6: miRNA families decreased in abundance in ΔTUT4/7 cells. Individual miRNA family members are significantly increased by at least 2-fold.

miRNA Family	DE-miRNA Count	miRNA Family Size	P-value	miRNA (significantly changed)
mir-34	3	3	1.06E-04	hsa-mir-34a, hsa-mir-34b, hsa-mir-34c
mir-204	2	2	2.26E-03	hsa-mir-204, hsa-mir-211
mir-105	2	2	2.26E-03	hsa-mir-105-1, hsa-mir-105-2
mir-24	2	2	2.26E-03	hsa-mir-24-1, hsa-mir-24-2
mir-23	2	2	2.26E-03	hsa-mir-23a, hsa-mir-23b

mir-3179	4	4	4.89E-06	hsa-mir-3179-1, hsa-mir-3179-2, hsa-mir-3179-3, hsa-mir-3179-4
mir-196	2	3	6.58E-03	hsa-mir-196a-1, hsa-mir-196a-2
mir-148	2	3	6.58E-03	hsa-mir-148a, hsa-mir-152
mir-450	2	3	6.58E-03	hsa-mir-450a-1, hsa-mir-450a-2
mir-8	3	5	9.86E-04	hsa-mir-200a, hsa-mir-200b, hsa-mir-429
mir-4421	1	2	0.0934	hsa-mir-4421
mir-216	1	2	0.0934	hsa-mir-216a
mir-192	1	2	0.0934	hsa-mir-215
mir-551	1	2	0.0934	hsa-mir-551b
mir-30	3	6	1.90E-03	hsa-mir-30a, hsa-mir-30c-1, hsa-mir-30c-2
mir-664	1	2	0.0934	hsa-mir-664a
mir-218	1	2	0.0934	hsa-mir-218-2
mir-10	3	8	4.97E-03	hsa-mir-125b-1, hsa-mir-125b-2, hsa-mir-99a

Supplementary Table S7: Group II miRNA decreased or increased by at least 2-fold in abundance, or unchanged in ΔTUT4/7 cells compared to WT cells.

	**Group 2 miRNA**
Overabundant in ΔTENT4A/B	hsa-let-7a-3p<br>hsa-let-7b-3p<br>hsa-let-7b-5p<br>hsa-let-7d-3p<br>hsa-let-7d-5p<br>hsa-let-7f-1-3p<br>hsa-let-7f-2-3p<br>hsa-let-7g-3p<br>hsa-let-7g-5p<br>hsa-let-7i-3p<br>hsa-let-7i-5p<br>hsa-miR-181d-5p<br>hsa-miR-1914-3p<br>hsa-miR-1914-5p<br>hsa-miR-365a-5p<br>hsa-miR-6765-3p<br>hsa-miR-6768-5p<br>hsa-miR-760<br>hsa-miR-98-3p<br>hsa-miR-98-5p
No change in ΔTENT4A/B<br>(fc between -2 ~ +2)	hsa-miR-1306-3p<br>hsa-miR-1306-5p<br>hsa-miR-130b-3p<br>hsa-miR-130b-5p

	hsa-miR-187-5p<br>hsa-miR-192-3p<br>hsa-miR-192-5p<br>hsa-miR-203a-3p<br>hsa-miR-23b-5p<br>hsa-miR-3065-3p<br>hsa-miR-3065-5p<br>hsa-miR-3146<br>hsa-miR-3173-3p<br>hsa-miR-3173-5p<br>hsa-miR-328-3p<br>hsa-miR-365a-3p<br>hsa-miR-421<br>hsa-miR-4483<br>hsa-miR-4484<br>hsa-miR-449b-5p<br>hsa-miR-551b-5p<br>hsa-miR-652-3p<br>hsa-miR-652-5p<br>hsa-miR-676-3p<br>hsa-miR-676-5p<br>hsa-miR-6761-5p<br>hsa-miR-6762-3p<br>hsa-miR-6764-5p<br>hsa-miR-6767-5p<br>hsa-miR-6769b-3p<br>hsa-miR-877-3p<br>hsa-miR-877-5p<br>hsa-miR-99b-3p<br>hsa-miR-99b-5p
Reduced abundance in ΔTENT3A/B	hsa-miR-143-3p<br>hsa-miR-187-3p<br>hsa-miR-23b-3p<br>hsa-miR-488-3p<br>hsa-miR-551b-3p<br>hsa-miR-99a-3p<br>hsa-miR-99a-5p

Supplementary Table S8: Gene Ontology (GO) - biological process analysis of mRNAs increased in abundance in ΔTUT4/7 cells.

GO biological process	REF-LIST	Upload	Expected	Fold Enrichment	Raw P-Value	False Discovery Rate
response to heat (GO:0009408)	98	11	2.52	4.36	9.37E-05	3.97E-02
rRNA processing (GO:0006364)	220	23	5.66	4.06	5.62E-08	1.47E-04
rRNA metabolic process (GO:0016072)	250	24	6.44	3.73	1.26E-07	1.97E-04
ribosome biogenesis (GO:0042254)	296	28	7.62	3.67	1.50E-08	1.18E-04
response to temperature stimulus (GO:0009266)	172	15	4.43	3.39	7.96E-05	3.46E-02
protein folding (GO:0006457)	219	18	5.64	3.19	3.29E-05	1.61E-02
ribonucleoprotein complex biogenesis (GO:0022613)	429	35	11.04	3.17	8.28E-09	1.30E-04
ncRNA processing (GO:0034470)	404	31	10.4	2.98	2.13E-07	2.78E-04
ncRNA metabolic process (GO:0034660)	524	34	13.49	2.52	2.60E-06	2.15E-03
RNA processing (GO:0006396)	858	45	22.09	2.04	1.58E-05	8.85E-03
cell-cell signaling (GO:0007267)	1055	50	27.16	1.84	6.32E-05	2.83E-02
regulation of transcription by RNA polymerase II (GO:0006357)	2619	103	67.42	1.53	1.77E-05	9.57E-03
regulation of nucleobase-containing compound metabolic process (GO:0019219)	4059	159	104.49	1.52	3.03E-08	9.49E-05
regulation of biosynthetic process (GO:0009889)	4155	162	106.96	1.51	2.92E-08	1.14E-04
regulation of RNA metabolic process (GO:0051252)	3753	146	96.61	1.51	2.10E-07	3.00E-04
regulation of molecular function (GO:0065009)	3079	119	79.26	1.5	6.67E-06	4.18E-03
regulation of biological quality (GO:0065008)	3730	144	96.02	1.5	4.86E-07	4.76E-04
regulation of cellular biosynthetic process (GO:0031326)	4095	158	105.41	1.5	8.64E-08	1.50E-04

regulation of macromolecule biosynthetic process (GO:0010556)	3932	150	101.22	1.48	4.60E-07	4.81E-04
regulation of transcription, DNA-templated (GO:0006355)	3455	131	88.94	1.47	4.45E-06	3.32E-03
regulation of nucleic acid-templated transcription (GO:1903506)	3456	131	88.96	1.47	4.47E-06	3.19E-03
regulation of RNA biosynthetic process (GO:2001141)	3465	131	89.2	1.47	5.91E-06	4.03E-03
regulation of primary metabolic process (GO:0080090)	5847	211	150.51	1.4	2.56E-08	1.34E-04
regulation of nitrogen compound metabolic process (GO:0051171)	5689	205	146.45	1.4	5.94E-08	1.16E-04
regulation of cellular metabolic process (GO:0031323)	6040	215	155.48	1.38	5.88E-08	1.32E-04
regulation of gene expression (GO:0010468)	4833	170	124.41	1.37	8.14E-06	4.91E-03
regulation of metabolic process (GO:0019222)	6720	231	172.99	1.34	2.34E-07	2.82E-04
cellular component organization or biogenesis (GO:0071840)	5659	192	145.67	1.32	1.46E-05	8.45E-03
regulation of macromolecule metabolic process (GO:0060255)	6190	210	159.34	1.32	4.03E-06	3.16E-03
regulation of cellular process (GO:0050794)	11275	348	290.24	1.2	5.37E-07	4.95E-04
regulation of biological process (GO:0050789)	11735	357	302.08	1.18	1.48E-06	1.29E-03
biological regulation (GO:0065007)	12469	378	320.98	1.18	4.05E-07	4.54E-04
biological_process (GO:0008150)	17859	490	459.72	1.07	5.00E-05	2.30E-02
Unclassified (UNCLASSIFIED)	2730	40	70.28	0.57	5.00E-05	2.37E-02
sensory perception of chemical stimulus (GO:0007606)	545	1	14.03	0.07	2.11E-05	1.07E-02

Supplementary Table S9: Gene Ontology (GO) - biological process analysis of mRNAs decreased in abundance in ΔTUT4/7 cells.

**GO biological process**	**REF-LIST**	**Upload**	**Expected**	**Fold Enrichment**	**Raw P-Value**	**False Discovery Rate**
regulation of fibroblast growth factor receptor signaling pathway (GO:0040036)	32	10	2.18	4.58	2.44E-04	2.99E-02
negative chemotaxis (GO:0050919)	46	13	3.14	4.14	7.07E-05	1.23E-02
autonomic nervous system development (GO:0048483)	48	12	3.27	3.67	3.50E-04	3.68E-02
neural crest cell migration (GO:0001755)	57	14	3.89	3.6	1.36E-04	2.06E-02
mesenchymal cell migration (GO:0090497)	59	14	4.02	3.48	1.85E-04	2.56E-02
antigen processing and presentation of exogenous peptide antigen (GO:0002478)	55	13	3.75	3.47	3.21E-04	3.47E-02
regulation of neural precursor cell proliferation (GO:2000177)	88	20	6	3.33	1.46E-05	3.41E-03
antigen processing and presentation of exogenous antigen (GO:0019884)	66	15	4.5	3.33	1.66E-04	2.37E-02
neural crest cell development (GO:0014032)	81	18	5.52	3.26	4.95E-05	9.58E-03
stem cell development (GO:0048864)	86	19	5.86	3.24	3.31E-05	7.01E-03
positive regulation of endothelial cell migration (GO:0010595)	107	22	7.3	3.02	2.16E-05	4.77E-03
endothelial cell differentiation (GO:0045446)	80	16	5.46	2.93	3.61E-04	3.75E-02
endothelium development (GO:0003158)	96	19	6.55	2.9	1.19E-04	1.89E-02
neural crest cell differentiation (GO:0014033)	93	18	6.34	2.84	2.27E-04	2.87E-02
cardiac chamber morphogenesis (GO:0003206)	124	23	8.46	2.72	5.82E-05	1.09E-02

regulation of endothelial cell migration (GO:0010594)	169	31	11.52	2.69	5.75E-06	1.64E-03
cell-matrix adhesion (GO:0007160)	133	23	9.07	2.54	1.80E-04	2.51E-02
morphogenesis of a branching epithelium (GO:0061138)	162	28	11.05	2.53	3.32E-05	6.94E-03
ear morphogenesis (GO:0042471)	129	22	8.8	2.5	2.87E-04	3.38E-02
morphogenesis of a branching structure (GO:0001763)	171	29	11.66	2.49	3.12E-05	6.71E-03
nephron development (GO:0072006)	130	22	8.86	2.48	3.08E-04	3.49E-02
regulation of osteoblast differentiation (GO:0045667)	126	21	8.59	2.44	4.92E-04	4.56E-02
branching morphogenesis of an epithelial tube (GO:0048754)	133	22	9.07	2.43	3.86E-04	3.90E-02
regulation of endothelial cell proliferation (GO:0001936)	134	22	9.14	2.41	4.17E-04	3.98E-02
positive regulation of epithelial cell migration (GO:0010634)	149	24	10.16	2.36	4.11E-04	3.97E-02
extracellular matrix organization (GO:0030198)	294	47	20.05	2.34	8.80E-07	3.28E-04
extracellular structure organization (GO:0043062)	295	47	20.12	2.34	9.07E-07	3.31E-04
stem cell differentiation (GO:0048863)	157	25	10.71	2.34	3.20E-04	3.48E-02
external encapsulating structure organization (GO:0045229)	297	47	20.25	2.32	9.76E-07	3.48E-04
cartilage development (GO:0051216)	161	25	10.98	2.28	4.00E-04	3.99E-02
cardiac chamber development (GO:0003205)	168	26	11.46	2.27	3.10E-04	3.50E-02
regulation of epithelial cell migration (GO:0010632)	228	35	15.55	2.25	3.54E-05	7.29E-03
cell-substrate adhesion (GO:0031589)	189	29	12.89	2.25	2.10E-04	2.77E-02
positive regulation of angiogenesis (GO:0045766)	163	25	11.12	2.25	4.53E-04	4.25E-02

positive regulation of vasculature development (GO:1904018)	163	25	11.12	2.25	4.53E-04	4.23E-02
negative regulation of Wnt signaling pathway (GO:0030178)	187	28	12.75	2.2	3.43E-04	3.64E-02
tube morphogenesis (GO:0035239)	666	99	45.42	2.18	1.52E-11	2.17E-08
regulation of cell-substrate adhesion (GO:0010810)	216	32	14.73	2.17	1.89E-04	2.60E-02
epithelial tube morphogenesis (GO:0060562)	311	46	21.21	2.17	5.39E-06	1.56E-03
angiogenesis (GO:0001525)	325	48	22.16	2.17	4.83E-06	1.43E-03
tube development (GO:0035295)	864	127	58.92	2.16	3.38E-14	5.29E-10
mesenchyme development (GO:0060485)	232	34	15.82	2.15	1.15E-04	1.88E-02
blood vessel morphogenesis (GO:0048514)	421	61	28.71	2.12	3.73E-07	1.67E-04
cell-cell adhesion via plasma-membrane adhesion molecules (GO:0098742)	264	38	18	2.11	6.83E-05	1.22E-02
morphogenesis of an epithelium (GO:0002009)	453	64	30.89	2.07	3.70E-07	1.70E-04
positive regulation of neurogenesis (GO:0050769)	220	31	15	2.07	3.89E-04	3.91E-02
muscle organ development (GO:0007517)	293	41	19.98	2.05	6.08E-05	1.12E-02
negative regulation of locomotion (GO:0040013)	336	47	22.91	2.05	1.83E-05	4.21E-03
negative regulation of cell motility (GO:2000146)	301	42	20.53	2.05	4.87E-05	9.55E-03
synapse organization (GO:0050808)	294	41	20.05	2.05	6.33E-05	1.15E-02
negative regulation of cellular component movement (GO:0051271)	309	43	21.07	2.04	3.97E-05	7.97E-03
vasculature development (GO:0001944)	530	73	36.14	2.02	1.71E-07	8.96E-05
tissue morphogenesis (GO:0048729)	560	77	38.19	2.02	6.11E-08	3.55E-05
negative regulation of cell migration (GO:0030336)	286	39	19.5	2	1.37E-04	2.03E-02

embryonic organ morphogenesis (GO:0048562)	301	41	20.53	2	1.19E-04	1.90E-02
blood vessel development (GO:0001568)	508	69	34.64	1.99	6.11E-07	2.52E-04
muscle structure development (GO:0061061)	480	65	32.73	1.99	1.20E-06	4.07E-04
positive regulation of nervous system development (GO:0051962)	268	36	18.28	1.97	3.35E-04	3.60E-02
wound healing (GO:0042060)	335	45	22.84	1.97	7.11E-05	1.22E-02
regulation of angiogenesis (GO:0045765)	285	38	19.43	1.96	3.11E-04	3.48E-02
positive regulation of cell development (GO:0010720)	296	39	20.18	1.93	2.71E-04	3.26E-02
regulation of vasculature development (GO:1901342)	289	38	19.71	1.93	3.50E-04	3.66E-02
anatomical structure formation involved in morphogenesis (GO:0048646)	913	120	62.26	1.93	1.53E-10	1.99E-07
circulatory system development (GO:0072359)	890	112	60.69	1.85	6.05E-09	4.52E-06
transmembrane receptor protein tyrosine kinase signaling pathway (GO:0007169)	408	51	27.82	1.83	1.43E-04	2.09E-02
animal organ morphogenesis (GO:0009887)	988	121	67.37	1.8	5.04E-09	4.16E-06
response to wounding (GO:0009611)	441	54	30.07	1.8	1.20E-04	1.87E-02
cell junction organization (GO:0034330)	487	59	33.21	1.78	8.24E-05	1.39E-02
gland development (GO:0048732)	406	49	27.69	1.77	3.11E-04	3.46E-02
regulation of cell migration (GO:0030334)	918	110	62.6	1.76	9.34E-08	5.23E-05
embryonic organ development (GO:0048568)	453	54	30.89	1.75	2.14E-04	2.80E-02
cell-cell adhesion (GO:0098609)	540	64	36.82	1.74	6.50E-05	1.17E-02
positive regulation of MAPK cascade (GO:0043410)	481	57	32.8	1.74	1.61E-04	2.32E-02

sensory organ development (GO:0007423)	574	68	39.14	1.74	3.75E-05	7.64E-03
positive regulation of cell migration (GO:0030335)	526	62	35.87	1.73	1.05E-04	1.75E-02
embryonic morphogenesis (GO:0048598)	586	69	39.96	1.73	4.33E-05	8.59E-03
positive regulation of cell adhesion (GO:0045785)	476	56	32.46	1.73	2.16E-04	2.79E-02
regulation of multicellular organismal development (GO:2000026)	1356	159	92.47	1.72	2.81E-10	3.15E-07
cell adhesion (GO:0007155)	964	113	65.74	1.72	1.80E-07	9.12E-05
regulation of anatomical structure morphogenesis (GO:0022603)	928	108	63.28	1.71	4.39E-07	1.86E-04
tissue development (GO:0009888)	1687	196	115.04	1.7	3.52E-12	7.89E-09
cell morphogenesis involved in differentiation (GO:0000904)	535	62	36.48	1.7	1.68E-04	2.37E-02
regulation of cell adhesion (GO:0030155)	773	89	52.71	1.69	6.63E-06	1.82E-03
regulation of cell development (GO:0060284)	496	57	33.82	1.69	3.84E-04	3.91E-02
regulation of cell motility (GO:2000145)	976	112	66.56	1.68	4.33E-07	1.88E-04
positive regulation of cell motility (GO:2000147)	550	63	37.51	1.68	2.04E-04	2.75E-02
enzyme-linked receptor protein signaling pathway (GO:0007167)	608	69	41.46	1.66	1.16E-04	1.87E-02
positive regulation of cellular component movement (GO:0051272)	564	64	38.46	1.66	2.42E-04	3.01E-02
heart development (GO:0007507)	538	61	36.69	1.66	3.37E-04	3.59E-02
positive regulation of locomotion (GO:0040017)	566	64	38.6	1.66	2.49E-04	3.02E-02
positive regulation of developmental process (GO:0051094)	1314	148	89.6	1.65	1.80E-08	1.13E-05
epithelium development (GO:0060429)	1054	118	71.87	1.64	7.63E-07	2.99E-04
cellular component morphogenesis (GO:0032989)	582	65	39.69	1.64	3.01E-04	3.49E-02

regulation of locomotion (GO:0040012)	1021	114	69.62	1.64	1.36E-06	4.52E-04
regulation of cellular component movement (GO:0051270)	1052	117	71.74	1.63	1.04E-06	3.63E-04
neurogenesis (GO:0022008)	1223	136	83.4	1.63	1.18E-07	6.39E-05
generation of neurons (GO:0048699)	1081	120	73.72	1.63	7.80E-07	2.98E-04
positive regulation of cell differentiation (GO:0045597)	863	95	58.85	1.61	1.85E-05	4.21E-03
anatomical structure morphogenesis (GO:0009653)	2185	240	149	1.61	1.83E-12	4.78E-09
regulation of cell differentiation (GO:0045595)	1568	172	106.92	1.61	4.76E-09	4.15E-06
embryo development (GO:0009790)	1044	113	71.19	1.59	5.76E-06	1.61E-03
central nervous system development (GO:0007417)	1000	108	68.19	1.58	9.31E-06	2.35E-03
cell morphogenesis (GO:0000902)	686	74	46.78	1.58	2.74E-04	3.28E-02
regulation of MAPK cascade (GO:0043408)	669	72	45.62	1.58	4.06E-04	3.97E-02
head development (GO:0060322)	804	86	54.83	1.57	1.11E-04	1.84E-02
neuron differentiation (GO:0030182)	1018	108	69.42	1.56	1.93E-05	4.32E-03
brain development (GO:0007420)	757	80	51.62	1.55	2.95E-04	3.44E-02
regulation of developmental process (GO:0050793)	2463	260	167.96	1.55	9.04E-12	1.57E-08
nervous system development (GO:0007399)	2081	218	141.91	1.54	1.27E-09	1.17E-06
neuron development (GO:0048666)	819	85	55.85	1.52	3.71E-04	3.80E-02
locomotion (GO:0040011)	1244	128	84.83	1.51	1.34E-05	3.29E-03
positive regulation of multicellular organismal process (GO:0051240)	1491	153	101.67	1.5	1.68E-06	5.48E-04
cell migration (GO:0016477)	873	89	59.53	1.5	4.29E-04	4.08E-02
localization of cell (GO:0051674)	1032	105	70.37	1.49	1.36E-04	2.05E-02
cell motility (GO:0048870)	1032	105	70.37	1.49	1.36E-04	2.03E-02

negative regulation of developmental process (GO:0051093)	898	91	61.24	1.49	4.04E-04	3.98E-02
regulation of cell population proliferation (GO:0042127)	1663	166	113.4	1.46	3.12E-06	9.96E-04
cell development (GO:0048468)	1668	166	113.74	1.46	3.25E-06	1.02E-03
regulation of protein phosphorylation (GO:0001932)	1108	110	75.56	1.46	2.23E-04	2.86E-02
system development (GO:0048731)	3719	367	253.61	1.45	4.91E-13	1.92E-09
multicellular organism development (GO:0007275)	4129	402	281.56	1.43	1.40E-13	7.29E-10
cell projection organization (GO:0030030)	1131	110	77.12	1.43	4.10E-04	3.99E-02
regulation of phosphorylation (GO:0042325)	1255	122	85.58	1.43	2.06E-04	2.76E-02
transmembrane transport (GO:0055085)	1204	117	82.1	1.43	3.05E-04	3.49E-02
animal organ development (GO:0048513)	3179	307	216.78	1.42	1.05E-09	1.03E-06
regulation of multicellular organismal process (GO:0051239)	2721	262	185.55	1.41	3.12E-08	1.88E-05
anatomical structure development (GO:0048856)	5029	472	342.94	1.38	9.47E-14	7.42E-10
positive regulation of signal transduction (GO:0009967)	1529	143	104.27	1.37	3.18E-04	3.49E-02
cell differentiation (GO:0030154)	3418	319	233.08	1.37	1.15E-08	7.86E-06
cellular developmental process (GO:0048869)	3440	321	234.58	1.37	1.02E-08	7.24E-06
regulation of intracellular signal transduction (GO:1902531)	1704	159	116.2	1.37	1.32E-04	2.04E-02
negative regulation of response to stimulus (GO:0048585)	1622	150	110.61	1.36	3.13E-04	3.45E-02
positive regulation of cell communication (GO:0010647)	1693	155	115.45	1.34	4.04E-04	4.01E-02
positive regulation of signaling (GO:0023056)	1698	155	115.79	1.34	4.15E-04	3.99E-02

developmental process (GO:0032502)	5561	505	379.21	1.33	1.36E-12	4.27E-09
regulation of localization (GO:0032879)	2762	250	188.35	1.33	8.09E-06	2.19E-03
regulation of cellular component organization (GO:0051128)	2345	209	159.91	1.31	1.25E-04	1.93E-02
regulation of signal transduction (GO:0009966)	2975	265	202.87	1.31	1.26E-05	3.13E-03
regulation of cell communication (GO:0010646)	3342	296	227.9	1.3	4.77E-06	1.44E-03
regulation of signaling (GO:0023051)	3355	295	228.78	1.29	8.33E-06	2.21E-03
cellular response to chemical stimulus (GO:0070887)	2599	227	177.23	1.28	2.03E-04	2.76E-02
positive regulation of cellular process (GO:0048522)	5740	482	391.42	1.23	3.56E-07	1.69E-04
regulation of biological quality (GO:0065008)	3730	310	254.36	1.22	2.77E-04	3.29E-02
multicellular organismal process (GO:0032501)	6472	536	441.34	1.21	2.50E-07	1.22E-04
regulation of response to stimulus (GO:0048583)	4001	331	272.84	1.21	2.33E-04	2.92E-02
negative regulation of cellular process (GO:0048523)	4867	399	331.89	1.2	6.91E-05	1.22E-02
positive regulation of biological process (GO:0048518)	6255	510	426.54	1.2	4.09E-06	1.26E-03
negative regulation of biological process (GO:0048519)	5288	429	360.6	1.19	7.34E-05	1.25E-02
cellular component organization (GO:0016043)	5459	437	372.26	1.17	2.09E-04	2.77E-02
cellular process (GO:0009987)	15176	1118	1034.88	1.08	6.32E-07	2.54E-04
biological_process (GO:0008150)	17859	1274	1217.84	1.05	9.06E-06	2.37E-03
Unclassified (UNCLASSIFIED)	2730	130	186.16	0.7	9.06E-06	2.33E-03
sensory perception (GO:0007600)	984	39	67.1	0.58	3.02E-04	3.48E-02
detection of stimulus (GO:0051606)	685	23	46.71	0.49	2.23E-04	2.84E-02

ribonucleoprotein complex biogenesis (GO:0022613)	429	9	29.25	0.31	2.87E-05	6.24E-03
detection of stimulus involved in sensory perception (GO:0050906)	559	8	38.12	0.21	1.42E-08	9.27E-06
sensory perception of chemical stimulus (GO:0007606)	545	7	37.16	0.19	5.33E-09	4.18E-06
detection of chemical stimulus (GO:0009593)	526	5	35.87	0.14	4.42E-10	4.62E-07
sensory perception of smell (GO:0007608)	470	3	32.05	0.09	2.30E-10	2.77E-07
detection of chemical stimulus involved in sensory perception (GO:0050907)	489	2	33.35	0.06	7.90E-12	1.55E-08
detection of chemical stimulus involved in sensory perception of smell (GO:0050911)	443	1	30.21	0.03	9.93E-12	1.56E-08

Supplementary Table S10: Target mapping in the miRNA/mRNA network. DE = significantly differentially expressed by at least 2-fold. Parentheses indicate number of target genes out of total number of target genes that are decreased in abundance in ΔTUT4/7 cells. Shared targets include mRNA targeted by multiple let-7 family miRNAs.

DE-miRNA	Target DE-mRNA decreased in abundance in ΔTUT4/7 cells
let-7a (32/639)	ARID3A, BMPR1B, C1RL, CASP8, CBX5, CDKN1A, DISC1, DTX3L, FAM13C, FMO4, HAND1, HMGA2, IKZF3, ITM2B, KIAA1328, KIAA2026, KREMEN1, KRTAP19-1, MFSD8, NEFM, NHLRC3, PAFAH2, PDE4DIP, PLD3, PMAIP1, SEL1L3, SESN1, SESN2, SLC19A3, TNFRSF10B, ZC3HAV1L, ZNF774
let-7b (48/1215)	ARID3A, ASIC1, BBS7, BTG2, C1RL, CACNG8, CAPG, CBX5, CDKN1A, DISC1, DLC1, DTX3L, EPB41L3, FMO4, HAND1, HMGA2, IKZF3, IPCEF1, JAG1, KCNC4, KIAA1328, KREMEN1, L3MBTL4, LRIG1, MFSD8, NDRG1, NEDD4, NHLRC3, NHSL1, PAAF1, PAFAH2, PLD3, PLXNB2, PMAIP1, PSD3, SEC11A, SLC19A3, SLC1A4, SMARCA1, SYNE1, TNFRSF10B, TNRC6B, UBA3, UTRN, VWA8, ZC3HAV1L, ZMYM2, ZNF774
let-7d (25/394)	ARID3A, ARRDC4, C1RL, CACNG8, CBR4, CBX5, CDKN1A, DISC1, DLC1, DTX3L, FMO4, HAND1, HMGA2, IKZF3, KIAA1328, KREMEN1, MFSD8, NHLRC3, PAFAH2, PLD3, PMAIP1, SLC19A3, TNFRSF10B, ZC3HAV1L, ZNF774
let-7f (23/397)	ARID3A, C1RL, CBX5, CDKN1A, DISC1, DTX3L, FMO4, FZD4, HAND1, HMGA2, IKZF3, KIAA1328, KREMEN1, MFSD8, NHLRC3, NKX2-1, PAFAH2, PLD3, PMAIP1, ROR2, SLC19A3, ZC3HAV1L, ZNF774

let-7g (23/341)	ARID3A, C1RL, CBX5, CDKN1A, DDR1, DISC1, DTX3L, FMO4, FN1, HAND1, HMGA2, IKZF3, KIAA1328, KREMEN1, MFSD8, NHLRC3, PAFAH2, PLD3, PMAIP1, SLC19A3, TNFRSF10B, ZC3HAV1L, ZNF774
let-7i (10/334)	ARID3A, C1RL, CBX5, CDKN1A, DISC1, DTX3L, FMO4, HAND1, HMGA2, IKZF3, KIAA1328, KREMEN1, MFSD8, NHLRC3, PAFAH2, PLD3, PMAIP1, SLC19A3, ZC3HAV1L, ZNF774
mir-98 (50/786)	ARHGAP28, ARID3A, ARMT1, ATP7B, C1RL, CALCB, CBX5, CDKN1A, CRYBG1, DISC1, DTX3L, ESRP2, FMO4, FOXO1, FRMD4B, FZD3, GCNT2, HAND1, HMGA2, IKZF3, INHBE, JAG1, KIAA1328, KLF3, KREMEN1, LEPROTL1, LRIG1, MFSD8, MITF, NARF, NHLRC3, OSMR, PAFAH2, PLAG1, PLAGL1, PLD3, PMAIP1, RASSF9, RHOBTB3, SALL4, SLC19A3, STC2, TFPI, TMEM164, TMEM45A, VAV3, VGLL3, ZC3HAV1L, ZFHX3, ZNF774
Shared targets	ARID3A, C1RL, CBX5, CDKN1A, DISC1, DTX3L, FMO4, HAND1, HMGA2, IKZF3, KIAA1328, KREMEN1, MFSD8, NHLRC3, PAFAH2, PLD3, PMAIP1, SLC19A3, ZC3HAV1L, ZNF774
